# Retromer Regulates Macro‐ and Micro‐Autophagy via Distinct Vacuolar Proteases in the Rice Blast Fungus

**DOI:** 10.1002/advs.202510068

**Published:** 2025-08-21

**Authors:** Dingyang Zhang, Jiexiong Hu, Yonghe Hong, Yuping Fan, Minghui Peng, Yakubu Saddeeq Abubakar, Shujing Liang, Nan Jiang, Lili Lin, Xiaohong Pan, Huakun Zheng, Naweed I. Naqvi, Zonghua Wang, Wenhui Zheng

**Affiliations:** ^1^ State Key Laboratory of Agricultural and Forestry Biosecurity & Key Lab of Biopesticide and Chemical Biology Ministry of Education College of Plant Protection Fujian Agriculture and Forestry University Fuzhou 350002 China; ^2^ Institute of Rice Research Fujian Academy of Agriculture Sciences Fuzhou 350003 China; ^3^ College of Life Sciences Fujian Agriculture and Forestry University Fuzhou 350002 China; ^4^ Temasek Life Sciences Laboratory and Department of Biological Sciences National University of Singapore Singapore 117604 Singapore; ^5^ Institute of Oceanography Minjiang University Fuzhou 350108 China

**Keywords:** macro‐autophagy, *Magnaporthe oryzae*, pexophagy, retromer complex, vacuolar proteases

## Abstract

The vacuole degrades and recycles endocytic and autophagic cargos, while the retromer complex sorts cargos from the endosomes to the trans‐Golgi network or the plasma membrane, thus preventing unnecessary vacuolar degradation. However, whether the retromer complex regulates vacuolar proteolytic system during autophagic substrate degradation remains unclear. This study demonstrates that the retromer complex regulates both general and selective autophagy by ensuring the delivery of vacuolar protease(s) into the vacuole lumen in the rice blast fungus *Magnaporthe oryzae*. The central retromer subunit, MoVps35, transports the serine protease MoPrb1 from the endosomes to the vacuole lumen. Deletion of *MoVPS35* or any other retromer component prevents the transport of MoPrb1‐GFP into the vacuole lumen. Consistently, Δ*Moprb1* mutant shows similar defects as the retromer mutants, including failure of autophagy‐dependent conidiation and plant infection. Additionally, mutation of the catalytic residues of MoPrb1 (Asp 192, His 224 and Ser 390) reduces autophagy flux. Furthermore, MoVps35 also interacts with another aspartyl protease MoPep4 via MoPrb1. Loss of *MoPEP4* leads to abnormal micro‐autophagy (pexophagy) but not to fungal development and pathogenicity. Overall, this study demonstrates a crucial role of the retromer complex in the regulation of macro‐ and micro‐autophagy by different vacuolar proteases in *M. oryzae*.

## Graphical abstract

In wild‐type of *M. oryzae* (Guy11), MoPrb1 and MoPep4 are delivered into the vacuole by the retromer complex to mediate protein degradation and selective autophagy. Their proper localization and catalytic functions are essential for the fungal vegetative growth, conidiation and pathogenicity. However, retromer mutations disrupt this transport, impairing conidial development and appressorium‐mediated host infection.

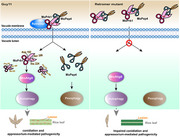

## 1 Introduction

Rice blast is a significant and persistent threat to rice production worldwide, causing immense damage to crops.^[^ ^1^, ^2^, ^3^ ^]^ The ascomycete fungus *Magnaporthe oryzae* causes the blast disease by invading rice tissue through the development of swollen and melanized infectious structures called appressoria.^[^ ^4^, ^5^ ^]^ Differentiation of an appressorium is controlled by the progression of the cell cycle, and later leads to autophagic cell death of the fungal conidial cells.^[^ ^6^, ^7^ ^]^ In *M. oryzae*, 22 autophagy (ATG) genes have been characterized and identified as components of the autophagic machinery.^[^ ^8^, ^9^ ^]^ Sixteen of these genes are required for non‐selective autophagy, also called macro‐autophagy, which is required for the regulated death of conidial cells and maturation of the appressorium.^[^ ^8^, ^10^ ^]^ Systematic deletion of these genes results in *M. oryzae* being non‐pathogenic, demonstrating that macro‐autophagy is essential for plant infection.^[^ ^6^, ^8^ ^]^ The remaining six genes are specifically associated with selective autophagy, such as pexophagy or mitophagy, and are dispensable for appressorium‐mediated plant infection.^[^ ^8^, ^11^, ^12^ ^]^ Therefore, understanding the role of autophagy in the rice blast fungus could unveil important drug targets for the control and management of the rice blast disease.

In addition, numerous studies have established a close link between the mechanisms of vesicle transport and the autophagy process in fungi. For example, the Rab GTPase Ypt7 (also known as Rab7) plays a conserved role in vesicle‐vacuole fusion during vacuole biogenesis in yeast and filamentous fungi.^[^ ^13^, ^14^, ^15^ ^]^ Deletion of the *YPT7* gene results in fragmented vacuoles, which impedes endocytosis and inhibits autophagy in *M. oryzae* and *Fusarium graminerarum*.^[^ ^15^, ^16^ ^]^ Mon1 is a potential guanine nucleotide exchange factor (GEF) for Ypt7, which activates Ypt7 in late endosomes and triggers endosomal maturation.^[^ ^17^, ^18^ ^]^ Deletion of *MoMON1* gene leads to defects similar to those observed in the Δ*Moypt7* mutant, including accumulation of small fragmented vacuoles in hyphal cells and blockage of autophagic pathways under both nutrient‐rich and starvation conditions.^[^ ^18^ ^]^ In addition, early endosomal transport proteins such as Rab5 and its GEF Vps9 are also involved in both endocytosis and autophagy in various phytopathogens.^[^ ^19^, ^20^, ^21^ ^]^ Recently, several studies have reported that components of the ESCRT (endosomal sorting complex required for transport) complex play crucial roles in the multivesicular body (MVB) sorting pathway, endocytosis and autophagy.^[^ ^22^, ^23^, ^24^ ^]^ Our previous study demonstrated the involvement of the retromer complex in regulating the autophagic process, which is indispensable for retrograde trafficking, development and autophagy‐dependent plant infection in *M. oryzae*.^[^ ^25^ ^]^ The retromer complex was first reported as a heteropentameric complex in yeast, consisting of Vps35, Vps26, Vps29, Vps17, and Vps5.^[^ ^26^ ^]^ It is known to participate in the intracellular retrograde transport of cargos from the endosome to the trans‐Golgi network.^[^ ^27^, ^28^ ^]^ We previously unveiled the role of MoVps17 in regulating endosome dynamics in *M. oryzae*, where we demonstrated that the retromer subcomplex MoVps17‐MoVps5 interacts directly with the endosomal membrane to mediate cargo sorting.^[^ ^29^ ^]^ Although deletion of *MoVPS35* leads to impaired autophagic cell death and autophagosome biogenesis in *M. oryzae*, ^[^ ^25^ ^]^ the detailed mechanisms by which MoVps35/retromer regulates autophagy and identifies its autophagy‐associated cargo proteins remain to be determined.

The process of autophagy is divided into several sequential steps including initiation, autophagosome formation and fusion with vacuoles or lysosomes, and delivery and degradation of substrates in the lumen.^[^ ^30^, ^31^ ^]^ However, the regulatory mechanisms involved in the degradation of substrates are not well understood.^[^ ^32^ ^]^ In yeast, the vacuole contains two major proteases: Prb1, which is a subtilisin‐like serine endopeptidase with a broad range of substrates, and Pep4, which is an aspartyl endopeptidase that cleaves preferentially between hydrophobic amino acids.^[^ ^33^, ^34^ ^]^ When both *PEP4* and *PRB1* are mutated, the degradation of proteins or autophagic vesicles is significantly impaired during nitrogen starvation, and sporulation is almost completely abolished.^[^ ^35^, ^36^ ^]^ In *M. oryzae*, the functions of Spm1, a Prb1 ortholog, have been analyzed.^[^ ^37^ ^]^ Spm1 is upregulated during appressorium formation and is necessary for invasion of leaf sheath in blast‐susceptible rice.^[^ ^38^, ^39^ ^]^ Deletion of the *SPM1* gene leads to accumulation of granular particles in conidial vacuoles, suggesting its role in degradation of autophagic bodies.^[^ ^37^ ^]^ The function of Pep4 in *M. oryzae* is currently unknown, but studies on its ortholog in *Ustilago maydis*, a phytopathogenic member of the Basidiomycota, have been conducted.^[^ ^40^ ^]^ Results showed that Pep4 is a vacuole‐localized proteinase and is important for *U. maydis* morphogenesis and virulence.^[^ ^40^ ^]^ While these studies have increased our knowledge of the vacuolar proteolytic system in autophagic substrate degradation, there are no reports that explore the relationship between the retromer complex and the endoproteases Prb1 and Pep4.

In this study, we used immunoprecipitation‐mass spectrometry (IP‐MS) and yeast two‐hybrid library screening to identify new retromer‐binding partners. Interestingly, the vacuole‐associated protease MoPrb1 was identified as one of the retromer‐interacting partners. Next, we utilized yeast two‐hybrid (Y2H) and bimolecular fluorescence complementation (BiFC) assays to confirm the direct interaction between MoVps35 and MoPrb1, and an indirect interaction between MoVps35 and MoPep4 via MoPrb1. Overall, MoVps35 delivers MoPrb1 to the vacuole lumen and this is important for autophagy‐dependent fungal morphogenesis and pathogenesis. Additionally, we found that through MoPrb1, MoVps35 also facilitates the transport of MoPep4 into the vacuole lumen for pexophagy, but this is dispensable for *M. oryzae* pathogenicity. Our results offer valuable insights into the mechanistic involvement of the retromer complex in both macro‐autophagy and micro‐autophagy in the rice blast fungus.

## 2 Results

### 2.1 MoVps35 Interacts with MoPrb1 on Endosomes

**Figure 1:**
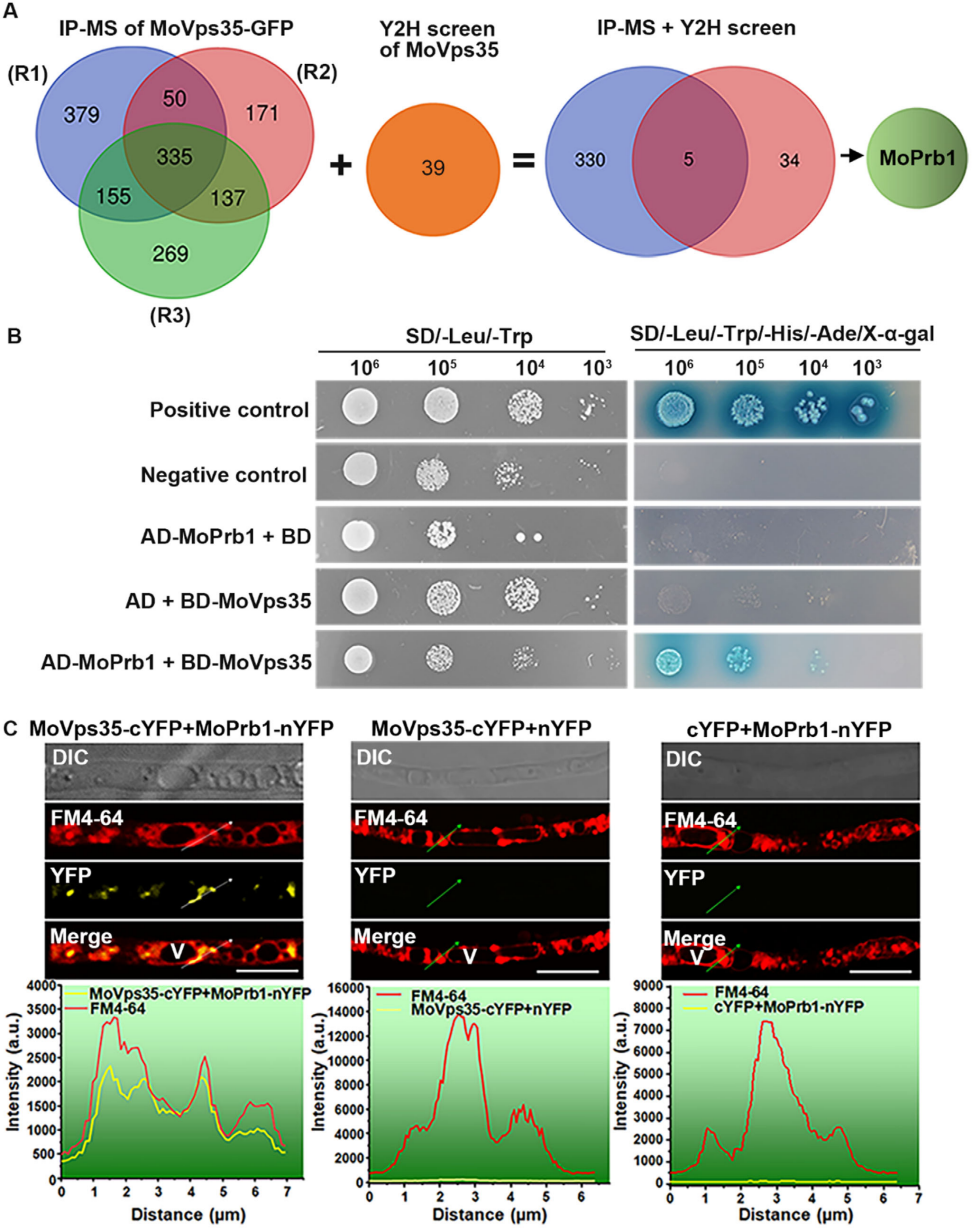
MoPrb1 interacts with MoVps35 on endosomes. A) A workflow showing the procedure adopted to identify MoPrb1 as MoVps35‐interacting partner. Two strategies were involved: MoVps35‐GFP affinity purification and mass spectrometric assays, and screening of *M. oryzae* cDNA library by MoVps35. B) Yeast two‐hybrid assays were performed to confirm the interaction between MoPrb1 and MoVps35. The MoVps35 bait (BD‐MoVps35) and MoPrb1 prey (AD‐MoPrb1) constructs were transformed into yeast cells. These transformants were then tested for growth on SD‐Leu‐Trp‐His‐Ade media supplemented with X‐α‐gal. Interaction between pGBKT7‐53 and pGADT7‐T served as a positive control, while that between pGBKT7‐Lam and pGADT7‐T served as a negative control. Various concentrations of the transformants (10^6^, 10^5^, 10^4^, and 10^3^ cells mL^−1^) were assayed. C) Bimolecular fluorescence complementation (BiFC) assay was used to visualize the interaction between MoVps35 and MoPrb1 in vivo. The yellow fluorescence signals were found in multiple puncta inside cell compartments and were highly co‐localized with the selective membrane dye FM4‐64. A line scan graph was generated at the indicated position (arrow) to show a positive interaction between MoVps35 and MoPrb1 on the FM4‐64‐labelled endosomes. Bar = 10 µm.

In an immunoprecipitation assay of cell lysates, we pulled MoVps35‐GFP using GFP‐Trap beads, followed by liquid chromatography‐tandem mass spectrometry (LC‐MS/MS) to identify retromer binding partners. From three biological replications, 335 potential interacting proteins were captured (**Figure** **1**A, Table S1, Supporting Information). Also, in a cDNA library screening, a total of 39 potential interactors were isolated (Figure 1A, Table S1, Supporting Information). To narrow down the number of MoVps35 interacting partners, we sorted out and analyzed only those proteins that were present in both the IP‐MS and cDNA library data. Thus, we ended up with a total of five new candidate interacting proteins (Table S1, Supporting Information). Among these proteins, MoPrb1 (MGG_03670) was identified as a subtilisin‐like proteinase and predicted to localize to the vacuolar compartment (Table S1, Supporting Information). Therefore, we chose MoPrb1 for further analyses.

Bioinformatic analysis showed that MoPrb1 is a 536‐amino‐acid protein and contains the peptidase inhibitor_I9 and serine peptidase_S8 domains (Figure S1A, Supporting Information). Phylogenetic analysis revealed that Prb1 is highly conserved through evolution and its orthologs are widely present in various fungi (Figure S1B, Supporting Information). To verify the physical interaction between MoVps35 and MoPrb1, a yeast two‐hybrid (Y2H) assay was conducted. We found that MoVps35 positively interacts with MoPrb1 (Figure 1B). To further confirm this interaction in vivo, we performed bimolecular fluorescence complementation (BiFC) analysis. The results showed reconstituted fluorescence signals that emerged as punctate structures in the fungal conidia, hyphae and appressoria in the strain coexpressing MoVps35‐cYFP and MoPrb1‐nYFP constructs, while no fluorescence signal was detected in the negative control (Figure S2, Supporting Information, Figure 1C). These data indicate that MoVps35 interacts with MoPrb1 both in vitro and in vivo. Our previous study demonstrated that MoVps35 mainly localizes to endosomes that are positioned close to the vacuolar membrane.^[^ ^25^ ^]^ To understand whether these puncta represent the endosomes, the fluorescent dye FM4‐64, which stains the plasma membrane, endosomes, and vacuolar membrane,^[^ ^41^, ^42^ ^]^ was used to trace the puncta observed in the BiFC assay in hyphae. The results revealed positive colocalization of the puncta with the endosomal membranes in the strains coexpressing MoVps35‐cYFP and MoPrb1‐nYFP (Figure 1C). Taken together, these data indicate that MoPrb1 interacts with MoVps35 on the endosomes.

### 2.2 MoPrb1 Is Important for Morphological Development of *M. oryzae*

To gain insights into the biological role of MoPrb1 in the developmental and pathogenic processes of the rice blast fungus, we generated *MoPRB1* gene deletion mutants in the wild‐type Guy11 strain, by replacing the entire open reading frame (ORF) with hygromycin B phosphotransferase cassette (*HPH*) (Figure S3A, Supporting Information). Candidate deletion mutants were verified by PCR and Southern blot (Figure S3B, Supporting Information). We also generated a complemented strain (Δ*Moprb1*‐com) by reintroducing the full‐length ORF of *MoPRB1* under the control of its native promoter into the Δ*Moprb1‐5* mutant (Table S2, Supporting Information).

**Figure 2:**
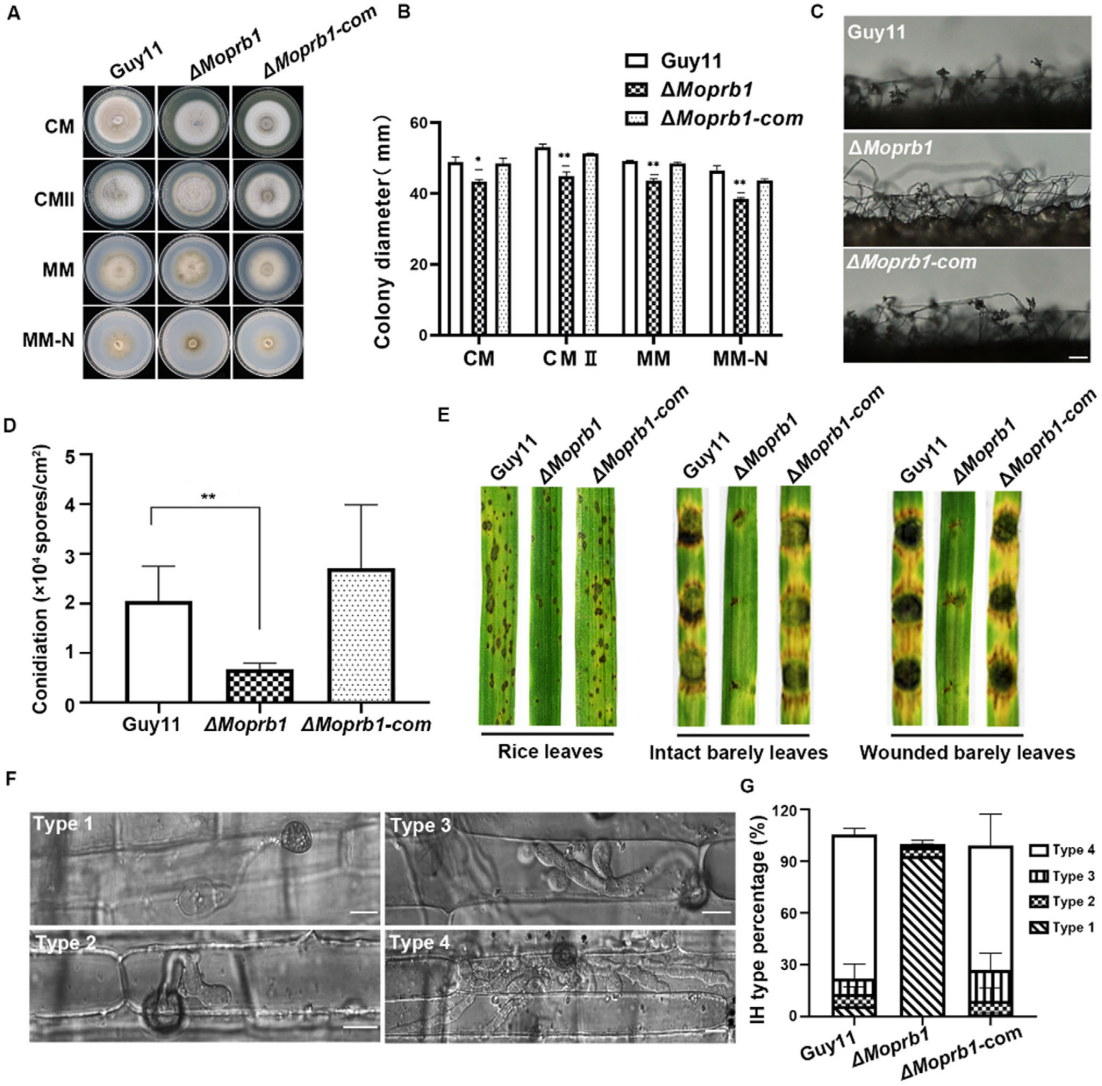
MoPrb1 is important for vegetative growth, conidiation, and pathogenicity of *M. oryzae*. A) MoPrb1 is required for vegetative growth. Colonies of the wild type (Guy11), the Δ*Moprb1* mutant, and the complemented strain (Δ*Moprb1*‐com) were grown on complete medium (CM), complete medium II (CM II), minimal medium (MM), and minimal medium without nitrogen (MM‐N, referred to as nitrogen starvation media) for 7 days. B) Analysis of colony diameters of the indicated strains grown on different media. Values are presented as mean ± standard deviation (SD) from three independent experiments. *p < 0.05, **p < 0.01. C) The development of conidia on conidiophores was significantly reduced in the Δ*Moprb1* mutant. Bar = 50 µm. D) Analysis of conidia production by the various strains. Values are presented as mean ± SD from three independent replicates, double asterisks indicate statistically significant difference (*p* < 0.01, n = 3). E) Pathogenicity assays on rice and barley leaves. The Δ*Moprb1* mutant showed defects in plant infection. The first panel (extreme left) shows intact rice leaves (*Oryza sativa* cv. CO39) sprayed with conidia suspensions obtained from the wild‐type strain (Guy11), the Δ*Moprb1* mutant and the complemented strain (Δ*Moprb1*‐com). Mycelial blocks of the indicated strains were inoculated on both intact and wounded barley leaves (second and third panels from the left, respectively) for 5 days. F,G) Rice leaf sheath penetration assay. Invasive hyphae (IH) growing in the rice cells were observed at 40 h post infection (hpi), and 4 types of IH were observed (type 1: appressorium formed but without IH, type 2: a single bulbous IH, type 3: branched bulbous IH but limited to one cell, type 4: IH spread to adjacent cells). Error bars represent standard deviations. A total of 50 invaded cells were analyzed, and the experiment was repeated thrice. Bar = 10 µm.

To investigate the role of MoPrb1 in the vegetative growth of *M. oryzae*, the wild‐type strain Guy11, the Δ*Moprb1* mutant and the complemented strain Δ*Moprb1*‐com were cultured on different nutrient‐sufficient (CM, CMII) and nutrient‐deficient (MM, MM‐N) media under the same incubation conditions (**Figure** **2**A). Such analyses showed that the growth rate of the Δ*Moprb1* mutant was lower than that of the wild type and complemented strains on the different culture media tested, indicating that MoPrb1 is involved in the normal vegetative growth of *M. oryzae* (Figure 2A,B). Conidiation is a crucial form of asexual reproduction in fungi.^[^ ^43^ ^]^ The Δ*Moprb1* mutant displayed a significant reduction in conidia/spore production compared to Guy11 and Δ*Moprb1*‐com cultured on RBA medium the number of conidia produced by the mutant was only 33% of that produced by the wild‐type strain (Figure 2C,D). Consistent with this result, we observed that the number of conidiophores as well as the number of conidia per conidiophore in the Δ*Moprb1* mutant were significantly reduced compared to the control strains (Figure 2C). In summary, these results suggest that MoPrb1 is important for vegetative growth and conidiogenesis in *M. oryzae*.

### 2.3 Deletion of *MoPRB1* Attenuates the Virulence of the Rice Blast Fungus

To elucidate the role of *MoPRB1* in the fungal infection process, we conducted pathogenicity assays by spraying conidia suspensions harvested from Guy11, Δ*Moprb1* and Δ*Moprb1*‐com strains onto the susceptible rice cultivar CO39 seedlings. The results showed that the number of disease lesions on the rice leaves infected with Δ*Moprb1* mutant was significantly reduced compared to the wild type (Figure 2E, left panel). Similar results were observed when both intact and injured barley leaves were inoculated with mycelial blocks from the CM cultures of the indicated strains (Figure 2E, middle and right panels).

Appressoria formation is crucial for successful host penetration and subsequent colonization by *M. oryzae*.^[^ ^44^ ^]^ We therefore hypothesized that the weakened virulence observed in the *MoPRB1* deletion mutant was due to malformation of appressoria. We therefore tested and compared the conidia germination as well as the appressorium formation abilities of the wild type strain Guy11, the Δ*Moprb1* mutant and the complemented strain Δ*Moprb1*‐com. This was achieved by incubating an equal number of conidia from the individual strains on hydrophobic coverslips at different time points. The results revealed a delayed conidial germination and appressorium formation in the Δ*Moprb1* mutant after 2 h of infection, when compared to the wild type and complemented strains (Figure S4, Supporting Information). At later time points, however, the Δ*Moprb1* mutant produced morphologically similar appressoria to those produced by the Guy11 and Δ*Moprb1*‐com strains (Figure S4, Supporting Information). Additionally, we also examined the role of MoPrb1 in turgor pressure accumulation and found that the collapse rates of Guy11 appressoria under 2 and 3 m glycerol concentrations were respectively 60% and 80% at 24 h post‐inoculation, whereas those in Δ*Moprb1* mutant were 75% and 98%, respectively, indicating a marked reduction in turgor pressure in the mutant strain (Figure S5, Supporting Information). These results indicate that deletion of *MoPRB1* delayed the early conidial germination (and hence appressoria formation) and caused abnormal appressorium turgor in *M. oryzae*.

Furthermore, rice leaf sheath penetration assays also showed that at 40 h post‐inoculation (Figure 2F,G), more than 80% of the appressoria formed by Guy11 and Δ*Moprb1*‐com successfully penetrated the rice cells and began to spread to neighboring cells. In contrast, ≈93% of the appressoria formed by the Δ*Moprb1* mutant were unable to breach the rice leaf cuticle. Although about 7% of the Δ*Moprb1* mutant appressoria were functional and successfully penetrated the initial rice leaf sheath cells, they were largely unable to further differentiate into invasive hyphae or extend to neighboring cells. Collectively, we conclude that targeted deletion of *MoPRB1* results in weakened pathogenicity of *M. oryzae* by perturbing the normal appressorial turgor, host penetration and invasive hyphal growth.

### 2.4 MoPrb1 Plays a Crucial Role in Autophagy

**Figure 3:**
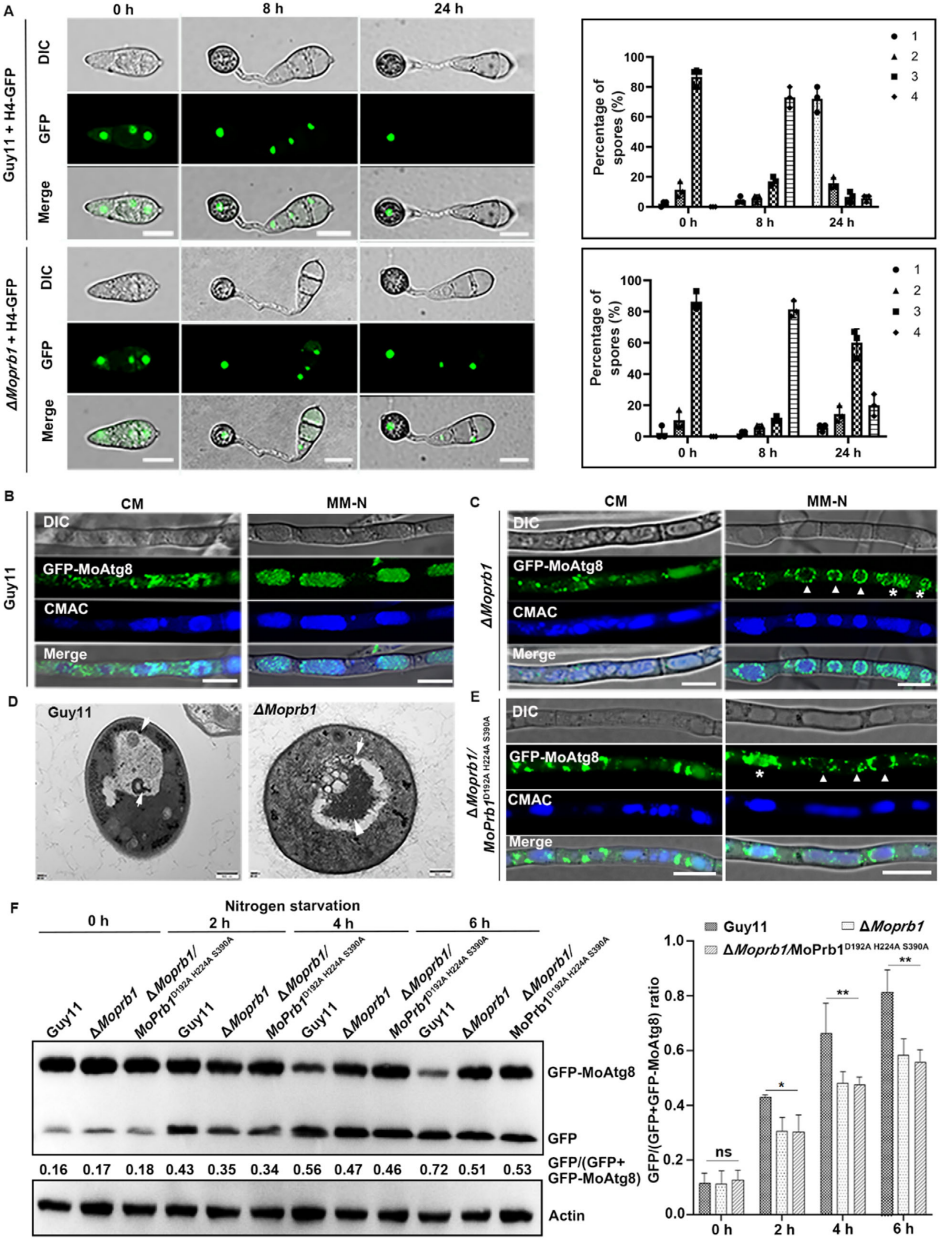
MoPrb1 is required for autophagy in *M. oryzae*. A) MoPrb1 is required for autophagy‐mediated conidial cell death during appressorium formation. Micrographs showing delayed nuclear degeneration in Δ*Moprb1* conidia during appressorium development. The bar charts on the right side show the percentage of spores in Guy11 strain and Δ*Moprb1* mutant containing 0 to 4 nuclei (n = 100, triple replicates) during appressorium development. Bar = 10 µm. B,C) Localization of GFP‐MoAtg8 in Guy11 and Δ*Moprb1* mutant cultured in liquid CM or MM‐N medium. Both the Guy11 and Δ*Moprb1* mutant strains expressing GFP‐MoAtg8 were grown in liquid CM medium at 28 °C for 24 h and then transferred to liquid MM‐N medium supplemented with 2 mm PMSF for 6 h to induce autophagy. Mycelia were stained with vacuole lumen dye CMAC (7‐amino‐4‐chloromethylcoumarin) and examined using a confocal microscope. Bar = 10 µm. Under autophagy‐induced conditions (MM‐N), many punctate GFP‐MoAtg8 failed to be delivered into the vacuoles for the effective operation of the autophagic process in Δ*Moprb1* strain (white arrowheads). Additionally, GFP‐MoAtg8‐labeled autophagosomes seemed unable to be degraded in the vacuoles due to the loss of *MoPRB1* gene (asterisk). D) Observation of autophagosome in the hyphal vacuoles of Guy11 and Δ*Moprb1* mutant by transmission electron microscopy. Vacuoles of the Guy11 hyphal cells were filled with autophagosomes (white arrows). In the Δ*Moprb1* mutant, as shown in the photographs, the fusion of the autophagosome with the endosome appears to be blocked, resulting in the accumulation of undegraded cellular contents in the vacuole (white arrows). Bar = 500 nm. E) Localization of GFP‐MoAtg8 in the Δ*Moprb1/*MoPrb1^D192A H224A S390A^ mutant cultured in liquid CM or MM‐N medium. The Δ*Moprb1/*MoPrb1^D192A H224A S390A^ mutant strains expressing GFP‐MoAtg8 were grown in liquid CM medium at 28 °C for 24 h and then transferred to liquid MM‐N medium supplemented with 2 mm PMSF for 6 h to induce autophagy. Mycelia were stained with vacuole lumen dye CMAC (7‐amino‐4‐chloromethylcoumarin) and examined using a confocal microscope. Bar = 10 µm. F) The Δ*Moprb1* mutant and Δ*Moprb1/*MoPrb1^D192A H224A S390A^ mutant showed a decrease in autophagy flux compared to the wild type. The Guy11, Δ*Moprb1*, and Δ*Moprb1/*MoPrb1^D192A H224A S390A^ mycelia were collected at each indicated time point after autophagy induction, and total protein was extracted for Western blot assay using anti‐GFP. Actin was used as an internal control. Quantification and comparison of autophagy flux from Western blot. GFP/(GFP+GFP‐MoAtg8) ratios were detected in Guy11 and Δ*Moprb1* mutant at 0, 2, 4, and 6 h. Band intensities of the GFP and GFP‐MoAtg8 were analyzed by ImageJ software (https://imagej.nih.gov/ij/). ^ns^ *p* > 0.05, *p < 0.05, **p < 0.01. Data are presented as mean ± SD, n = 3.

Previous studies demonstrated the important role of MoVps35 in autophagy‐mediated conidial cell death during appressorium formation.^[^ ^25^, ^45^ ^]^ Additionally, Prb1 from *Alternaria alternata* has been shown to participate in autophagy process.^[^ ^46^ ^]^ Therefore, we investigated whether MoPrb1 is also involved in autophagy during conidial cell death in *M. oryzae*. Histone 4 (H4‐GFP) fusion construct (a nuclear marker) was introduced into the wild type and Δ*Moprb1* mutant strains to check for nuclear degeneration during appressorium formation. Similar to a previous observation in Δ*Movps35* mutant,^[^ ^25^ ^]^ the nuclear degeneration in conidia was significantly retarded in the Δ*Moprb1* during appressorium formation (**Figure** **3**A). To further validate that this defect is associated with autophagy deficiency, we included a Δ*Moatg8* mutant as a control (Figure S6 Supporting Information), which also exhibited similar nuclear degradation delay. Consistently, glycogen and lipid bodies mobilization from conidia to appressoria were also obviously delayed in the Δ*Moprb1* mutant (Figures S7 and S8, Supporting Information). In previous studies, nuclear degradation has been shown to be associated with ferroptosis in *M. oryzae*.^[^ ^47^ ^]^ To further test whether this process is associated with ferroptosis, we performed C11‐BODIPY^581/591^ staining assays according to previous studies.^[^ ^47^ ^]^ The results showed no significant differences in lipid peroxidation levels between the Δ*Moprb1* mutant and the wild‐type strain (Figure S9, Supporting Information), suggesting that the defect of Δ*Moprb1* in nuclear degradation is independent of ferroptosis. Collectively, these data suggest the involvement of MoPrb1 in the autophagy‐mediated conidial cell death during appressorium formation.

To further understand the role of MoPrb1 in autophagy, we compared the localization of GFP‐MoAtg8 (an epifluorescent marker of autophagosomes) in Guy11 and Δ*Moprb1* mutant strains cultured in liquid CM or MM‐N medium. In Guy11, we observed that GFP‐MoAtg8 majorly appears as punctate structures in the cytosol of the hyphae grown in the nutrient‐rich medium (liquid CM) for 48 h; however, when these hyphae were shifted to nitrogen starvation medium (liquid MM‐N medium) containing 2 mM PMSF (phenylmethylsulfonyl fluoride) and grown for another 6 h, the GFP‐MoAtg8 signals were evident in the vacuole lumen (Figure 3B), indicating the occurrence of autophagy. On the other hand, in Δ*Moprb1* mutant hyphae, most of the GFP‐Atg8 signals remained punctate but most of them failed to enter the vacuoles after induction of autophagy. Additionally, we found that a few GFP‐MoAtg8‐labeled autophagosomes that appeared in the vacuoles remained undegraded in the vacuole of the mutant (Figure 3C). Moreover, the ultrastructures of autophagosomes in the hyphal vacuoles of both Guy11 and Δ*Moprb1* strains were further analyzed by transmission electron microscopy (TEM). As shown in Figure 3D, in the Δ*Moprb1* mutant, some autophagosome‐like structures accumulated in the vacuole lumen and remained attached to the rim of the vacuole. In addition, many undigested subcellular structures with strong electron density were repeatedly found in the Δ*Moprb1* mutant. To further explore whether the MoAtg8 degradation was associated with MoPrb1 serine protease activity, we introduced site‐directed mutations to inactivate its catalytic residues (Asp 192, His 224 and Ser 390)^[^ ^48^ ^]^ and subsequently analyzed the degradation of MoAtg8‐GFP in vivo. We observed that in the Δ*Moprb1/*MoPrb1^D192A H224A S390A^ mutant, the GFP‐Atg8 signals exhibit a distribution pattern similar to that of the Δ*Moprb1* mutant (Figure 3E), suggesting that the enzyme activity of MoPrb1 is required for the normal degradation of the MoAtg8 protein. To further confirm this, total protein was extracted from Guy11, Δ*Moprb1* and Δ*Moprb1/*MoPrb1^D192A H224A S390A^ strains cultured in MM‐N media at different time points, and subjected to Western blot analysis. The results showed an increasing abundance of GFP with increase in autophagy induction time in the Guy11 strain, with a corresponding decrease in the abundance of GFP‐MoAtg8. However, in the Δ*Moprb1* and Δ*Moprb1/*MoPrb1^D192A H224A S390A^ strains, the abundance of GFP and GFP‐MoAtg8 remained very similar (Figure 3F). By quantifying the ratio of GFP/(GFP+GFP‐MoAtg8) in Guy11, Δ*Moprb1* and Δ*Moprb1/*MoPrb1^D192A H224A S390A^ mutants at different time points, it was found that the ratio increased to 0.72 in Guy11 after 6 h of nitrogen starvation while this ratio only increased to 0.51 and 0.53 in the Δ*Moprb1* and Δ*Moprb1/*MoPrb1^D192A H224A S390A^ mutants, indicating that autophagy flux was remarkably affected in the mutants (Figure 3F). Collectively, these results indicate that MoPrb1 not only plays a crucial role in autophagy‐based conidial cell death, but is also involved in autophagy‐dependent degradation pathway in *M. oryzae*.

### 2.5 MoPrb1 Mainly Localizes to Vacuoles and Co‐Localizes with MoVps35 Outside the Vacuoles

**Figure 4:**
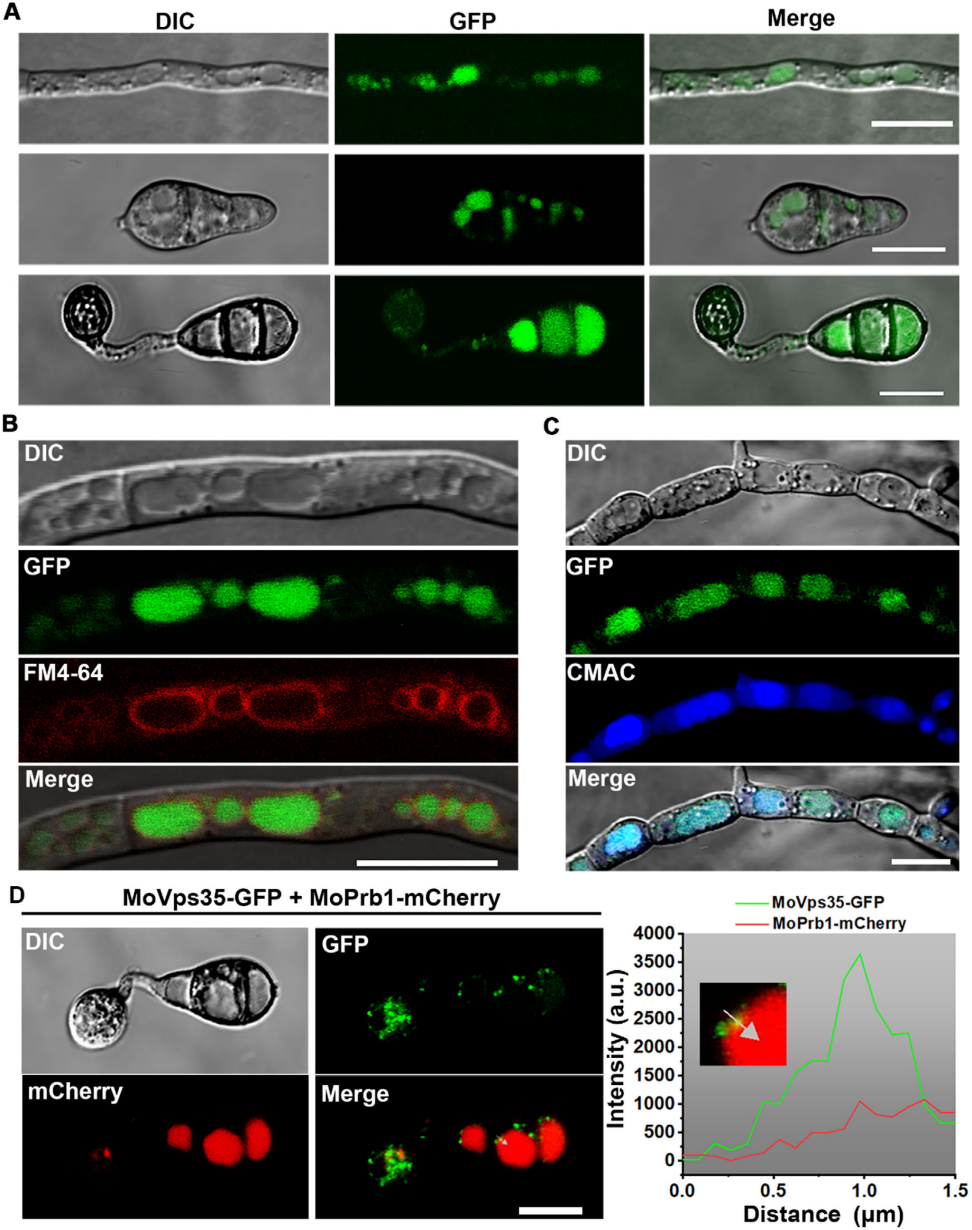
Subcellular localization of MoPrb1‐GFP fusion protein. A) Confocal microscopic examination for localization of MoPrb1‐GFP fusion protein at different developmental stages. MoPrb1 is observed to be expressed mainly in the vacuole lumen, with a few fluorescence observed in some punctate structures in hyphae, conidia, and germinal conidia. Bar = 10 µm. B) Mycelia expressing MoPrb1‐GFP were stained with FM4‐64 (a vacuolar membrane dye) and examined using confocal microscope. Bar = 10 µm. C) Mycelia expressing MoPrb1‐GFP were stained with CMAC (7‐amino‐4‐chloromethylcoumarin, a vacuolar lumen dye) and examined using a confocal microscope. Bar = 10 µm. D) MoPrb1‐mCherry colocalizes with MoVps35‐GFP in conidia. The line scan graphs confirm the colocalization of MoVps35‐GFP and MoPrb1‐mCherry at the punctate structures close to the vacuole. Bar = 10 µm.

In *Saccharomyces cerevisiae*, Prb1 has been reported to participate in protein degradation in the vacuole.^[^ ^49^ ^]^ However, recent studies have revealed that Prb1 has additional functions beyond its role in protein degradation.^[^ ^50^ ^]^ It can exit the vacuole and localize to other areas within the cell, where it performs H3 clipping to alter gene expression and inhibit prion formation.^[^ ^50^, ^51^, ^52^ ^]^ Therefore, to monitor the subcellular localizations of MoPrb1 at different developmental stages of the rice blast fungus, we used a laser scanning confocal microscope to examine the hyphae, conidia and appressoria produced by the Δ*Moprb1*‐com strain expressing MoPrb1‐GFP fusion construct. At the various developmental stages of the fungus, the MoPrb1‐GFP fusion protein was observed in some large compartments presumed to be vacuoles, with a few fluorescence observed as punctate structures outside these compartments (**Figure** **4**A). To confirm that these compartments are actually vacuoles, the endosome/vacuole membrane dye FM4‐64 and the vacuole‐specific dye CMAC (7‐amino‐4‐chloromethylcoumarin) were used to stain the fungal hyphae. As shown in Figure 4B,C, the MoPrb1‐GFP fluorescence clearly co‐localized with the CMAC fluorescence, surrounded by FM4‐64 fluorescence signal, confirming the localization of MoPrb1 in the vacuole lumen. Furthermore, we reasoned that the punctate structures outside the vacuoles could be endosomes. Since the retromer complex localizes to the endosomes,^[^ ^25^, ^29^ ^]^ we decided to test this by investigating the possible co‐localization of MoPrb1‐mCherry with the core retromer subunit MoVps35‐GFP. Interestingly, the MoPrb1‐mCherry co‐localized with the MoVps35‐GFP on the punctate structures near the vacuoles in conidia, further supporting the above identified interactions between the two proteins on endosomes (Figure 4D). Thus, we conclude that MoPrb1 primarily localizes to the vacuole lumen at all developmental stages of *M. oryzae*, and co‐localizes and interacts with the retromer complex on endosomes.

### 2.6 The Retromer Complex Components Are Essential for the Vacuole Lumen Localization of MoPrb1

**Figure 5:**
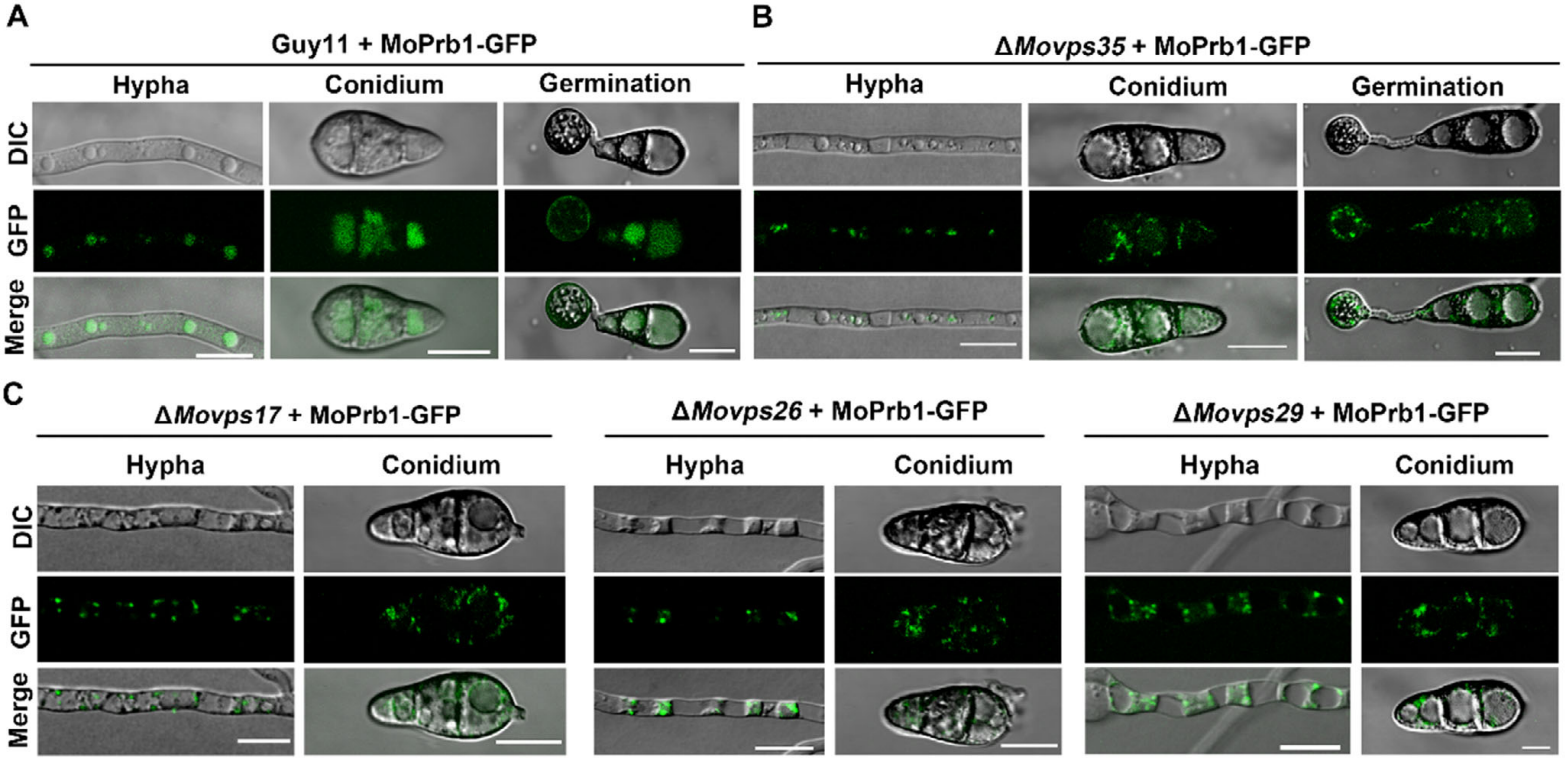
The retromer complex components are required for the correct localization of MoPrb1‐GFP. A) In Guy11, the subcellular localization of MoPrb1‐GFP is mainly observed in the vacuole lumen at different developmental stages, including hyphae, conidia, and during conidia germination. B) Subcellular localization of MoPrb1‐GFP in the Δ*Movps35* mutant. Deletion of *MoVPS35* gene, a core component of the retromer complex, caused MoPrb1 mis‐localization to the vacuole lumen at the different developmental stages of the fungus. C) Subcellular localization of MoPrb1‐GFP in Δ*Movps17*, Δ*Movps26*, and Δ*Movps29* mutant strains, respectively. *MoVPS26*, *MoVPS29*, and *MoVPS17* play a role in the correct localization of MoPrb1‐GFP. Bar = 10 µm.

Since MoVps35 interacts with MoPrb1, and the retromer complex is known to regulate the retrograde transport of vesicles, we hypothesized that the retromer may mediate the transport of MoPrb1 to the vacuole. To validate this, we checked the localization of MoPrb1‐GFP in both the wide type (Guy11) and Δ*Movps35* mutant. Laser confocal microscopy revealed that MoPrb1‐GFP exhibited its normal vacuole lumen localization in the wild type (**Figure** **5**A) but displayed punctate localization outside the vacuole in Δ*Movps35* mutant (Figure 5A,B), suggesting that MoVps35 is required for the delivery of MoPrb1 to the vacuole lumen. Similarly, we checked the localization of MoPrb1‐GFP in the absence of the other subunits (MoVps17, MoVps26, MoVps29 and MoVps5) of the retromer complex^[^ ^25^, ^29^ ^]^ and found a similar result to that observed in the Δ*Movps35* mutant (Figure 5C). Upon close observation, we noticed that most of these punctate structures were positioned close to the vacuole membrane (Figure 5B,C). Furthermore, to ascertain whether the deletion of *MoPRB1* also impacts the subcellular localization of MoVps35, a fusion protein of MoVps35 with mScarlet was introduced into the wild type and Δ*Moprb1* mutant. Results obtained from microscopy assays showed that the distribution of MoVps35‐mScarlet was similar in both Guy11 and the Δ*Moprb1* mutant (Figure S10, Supporting Information), suggesting that MoPrb1 does not influence the subcellular localization of MoVps35. Taken together, these data support that the retromer complex serves as a key machinery for the delivery of MoPrb1 to the vacuole lumen in *M. oryzae*.

### 2.7 MoVps35 Interacts with the Aspartyl Protease MoPep4 via MoPrb1

**Figure 6:**
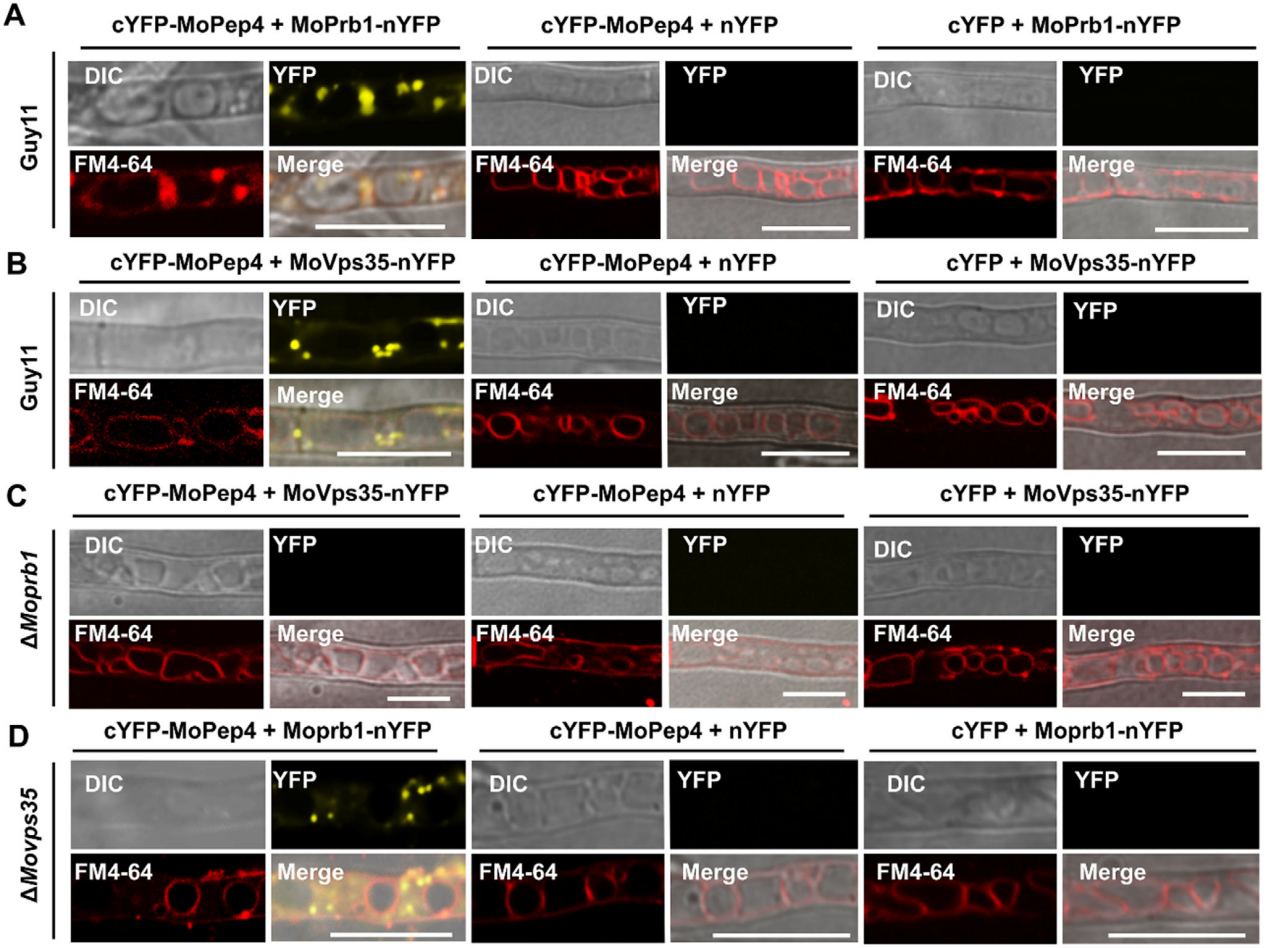
The vacuolar aspartyl protease MoPep4 directly interacts with MoPrb1 and indirectly with MoVps35 via MoPrb1. A,B) Biomolecular fluorescence complementation (BiFC) assay was used to visualize the interactions between MoVps35, MoPrb1 and MoPep4 in vivo. In the Guy11, reconstituted YFP signals were observed in strains expressing the cYFP‐MoPep4/MoPrb1‐nYFP constructs as well as in strains expressing the cYFP‐MoPep4/MoVps35‐nYFP constructs. However, C) in the Δ*Moprb1* mutant, no reconstituted YFP signal was detected in strains expressing the cYFP‐MoPep4/MoVps35‐nYFP constructs. D) In the Δ*Movps35* mutant, reconstituted YFP signals were observed in strains expressing the cYFP‐MoPep4/MoPrb1‐nYFP constructs, while YFP signals were not detected in the negative control strains. Bar = 10 µm.

In *S. cerevisiae*, the vacuolar proteolytic system consists of at least seven luminal proteases, including PrA, PrB, CpY, CpS, ApI, ApY and DPAP‐B.^[^ ^53^, ^54^ ^]^ The vacuolar protease genes *PRB1* (encoding the serine endoprotease PrB) and *PEP4* (encoding the aspartyl endoprotease PrA) actively participate in activating a range of vacuolar hydrolases, thus playing an important role in autophagy.^[^ ^55^ ^]^ Pep4 is an aspartyl protease that requires Prb1 for its activation, while the precursor of Prb1 also requires Pep4 for the cleavage of its C‐terminal peptide.^[^ ^32^, ^54^ ^]^ Interestingly, we revisited our MoVps35 IP‐MS data and found MoPep4 (MGG_00922) as a potential interacting partner of MoVps35, although the hit number is less than that of MoPrb1 (Table S1, Supporting Information). Therefore, we checked for direct interaction between MoPep4 and MoVps35 by Y2H but found no interaction between the two proteins (Figure S11, Supporting Information). However, the BiFC assay indicated that MoPep4 interacts with MoVps35 (**Figure** **6**B), suggesting an indirect interaction between these two proteins. Therefore, we used BiFC analysis to determine whether the interaction between MoPep4 and MoVps35 depends on MoPrb1 and found that the interaction of MoVps35 and MoPep4 was blocked in the absence of *MoPRB1* (Figure 6C). These findings confirm our hypothesis that MoPep4 and MoVps35 interact indirectly via MoPrb1 (Figure 6 and Figure S12, Supporting Information). Moreover, we found that the interaction between MoPrb1 and MoPep4 remains unaffected in the absence of *MoVPS35* (Figure 6A,D). To elucidate how MoPrb1 regulates the interaction between MoPep4 and MoVps35, we examined the subcellular localization of GFP‐MoPep4 in the Δ*Moprb1* mutant and Moprb1‐GFP in the Δ*Mopep4* mutant. Our results showed that GFP‐MoPep4 localized on the vacuolar membrane but not in the vacuole lumen in the Δ*Moprb1* mutant. In contrast, the absence of *MoPEP4* had little effect on the MoPrb1‐GFP localization to vacuole lumen, with only a fraction forming punctate structures on the vacuolar membrane (Figure S13A, Supporting Information). Western blot analysis showed that MoPep4 could not be properly degraded in the Δ*Moprb1* mutant, suggesting that MoPrb1 regulates MoPep4 to ensure its normal localization (Figure S13B, Supporting Information). Collectively, these results indicate that MoPrb1 regulates MoPep4 maturation and subsequently mediates its interaction with the retromer complex in *M. oryzae*.

### 2.8 The Retromer Complex Is Required for MoPep4 Localization to the Vacuole Lumen

**Figure 7:**
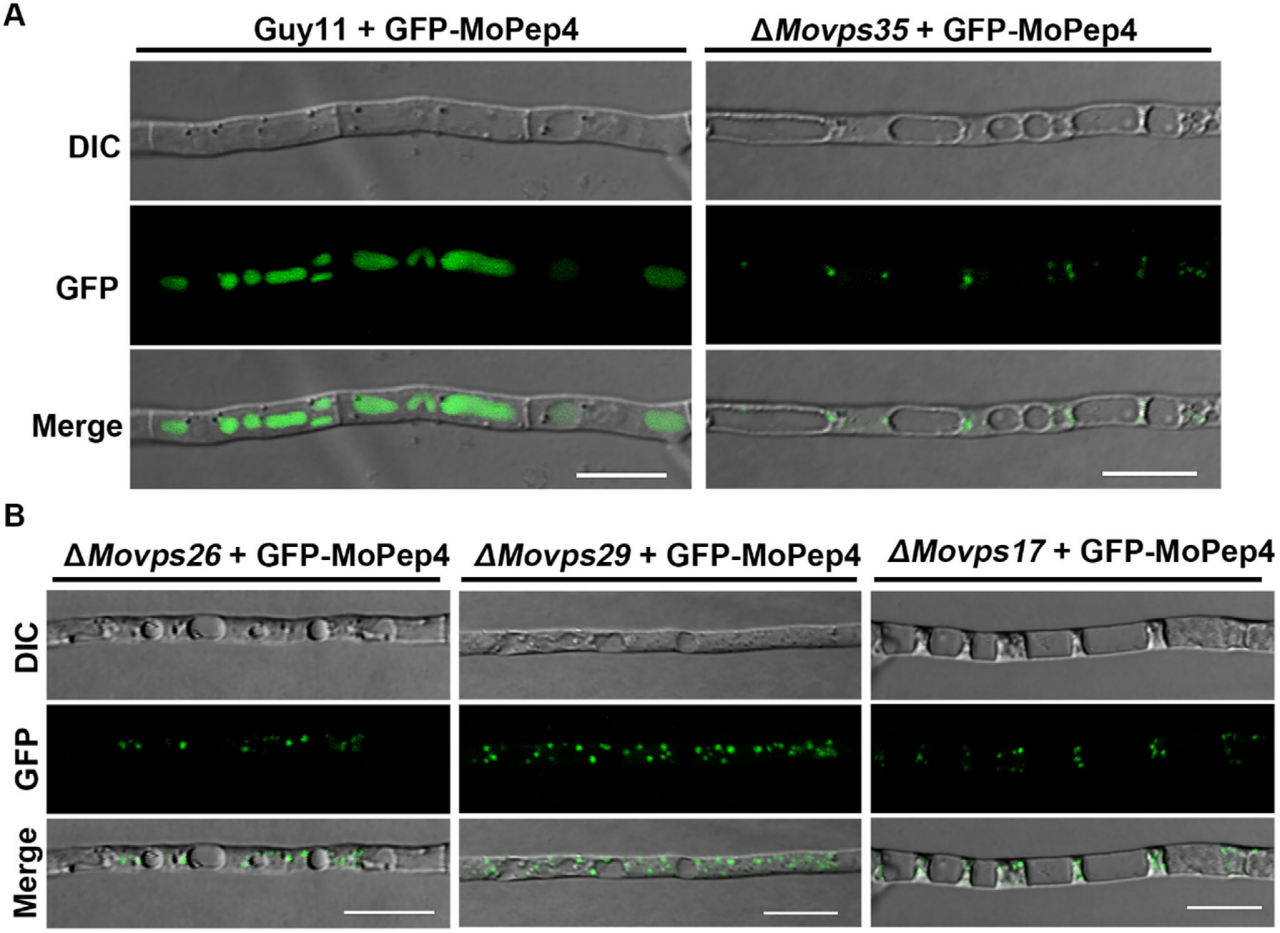
The retromer complex components are required for the correct localization of GFP‐MoPep4. A) Subcellular localization of MoPep4‐GFP in Guy11 and Δ*Movps35* mutant. GFP‐MoPep4 localizes in vacuoles in Guy11. Deletion of the *MoVPS35* gene in Guy11 causes MoPrb1‐GFP mis‐localization to the vacuoles. B) Subcellular localization of GFP‐MoPep4 in the Δ*Movps26*, Δ*Movps29*, and Δ*Movps17* mutants, respectively. The images indicate that *MoVPS26*, *MoVPS29*, and *MoVPS17* may also be involved in the vacuole lumen localization of GFP‐MoPep4. Bar = 10 µm.

To further understand the link between the retromer complex and MoPep4 in *M. oryzae*, we first investigated the subcellular localization of MoPep4. Confocal microscopy was performed on Guy11 strain expressing GFP‐MoPep4 fusion construct, with the vacuole lumen and membrane stained using the fluorescent dyes CMAC and FM4‐64, respectively. The results revealed that the GFP‐MoPep4 fluorescence signal was mainly concentrated in the vacuole lumen, co‐localizing with CMAC fluorescence and surrounded by FM4‐64 fluorescence (**Figure** **7**A, left panel and Figure S14B,C, Supporting Information). In the mutants of the retromer subunits (Δ*Movps35*, Δ*Movps17*, Δ*Movps26* and Δ*Movps29*), GFP‐MoPep4 was only detected in punctate vesicles and failed to localize to the vacuole lumen of each mutant strain (Figure 7A, right panel,B). Altogether, these results suggest that the retromer complex is indispensable for the localization of MoPep4 to the vacuole lumen.

### 2.9 MoPep4 Is Dispensable for Vegetative Growth, Conidiation, Pathogenicity and Atg8‐Related Autophagy in *M. oryzae*

**Figure 8:**
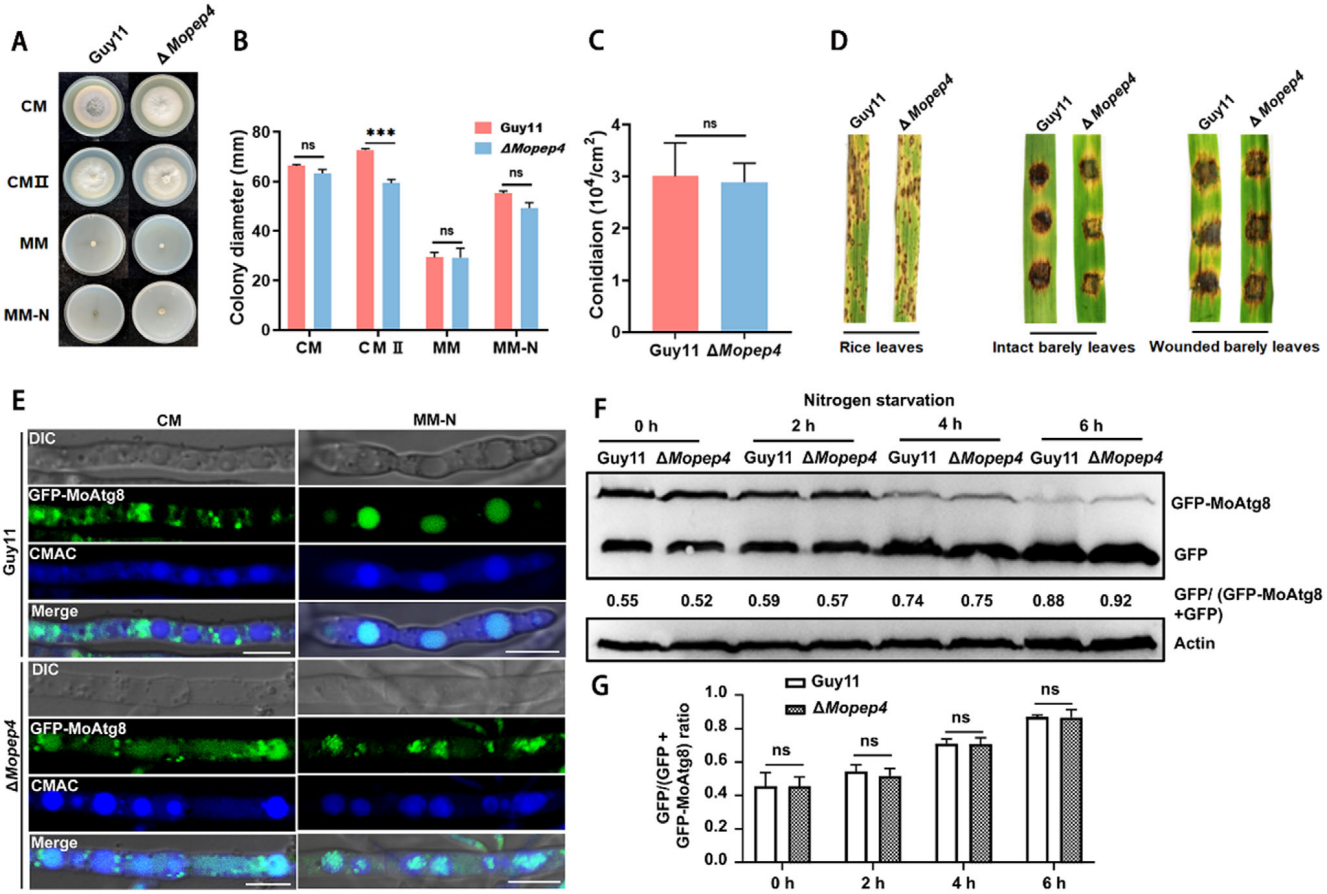
MoPep4 is dispensable for mycelial growth, conidiation, pathogenicity, and autophagy in *M. oryzae*. A) Deletion of *MoPEP4* does not show any obvious defects in vegetative growth. Colonies of Guy11 and Δ*Mopep4* mutant were grown on CM, CM II, MM, and MM‐N media for 7 days. B) Analysis of colony diameters of the indicated strains grown on different media. Values are presented as mean ± SD from three independent replicates. ^ns^ *p* > 0.05, ***p < 0.001. C) Analysis of conidia production by the indicated strains. Values are presented as mean ± SD from three independent replicates. ^ns^ *p* > 0.05. D) Deletion of *MoPEP4* does not attenuate the fungal pathogenicity on barley leaves and rice seedlings. Seedlings of the susceptible rice cultivar CO39 were inoculated with conidia suspensions (1 × 10^5^ conidia mL^−1^) obtained from the indicated strains and incubated for 7 days. E) Subcellular localization of GFP‐MoAtg8 in Guy11 and Δ*Mopep4* mutant grown on CM or MM‐N medium. Deletion of *MoPep4* does not affect the subcellular localization of GFP‐MoAtg8. Vacuoles were stained with CMAC. F,G) Quantification and comparison of autophagy flux in Guy11 and Δ*Mopep4* mutant by Western blot analysis. Mycelia from Guy11 and Δ*Mopep4* mutant were collected at the indicated time points after autophagy induction, and total protein was extracted for Western blot assay using anti‐GFP. Actin was used as an internal control. GFP/(GFP+GFP‐MoAtg8) ratios were detected in Guy11 and Δ*Mopep4* mutant at 0 and 6 h. Bar = 10 µm. Band intensities of the GFP and GFP‐MoAtg8 were analyzed by ImageJ software (https://imagej.nih.gov/ij/). ^ns^ *p* > 0.05. Data are presented as mean ± SD, n = 3.

Previous studies have demonstrated the importance of Pep4 in maintaining the functionality of vacuolar proteolytic system in *S. cerevisiae*, and its involvement in dimorphism and pathogenesis of *U. maydis*.^[^ ^40^, ^56^ ^]^ Therefore, we set to unveil the biological functions of MoPep4 in the rice blast fungus. First, we generated deletion mutants of *MoPEP4* and confirmed the deletion by Southern blot assay (Figure S15, Supporting Information). Guy11 and the Δ*Mopep4* mutant were subsequently grown on CM, CM II, MM and MM‐N media for 7 days under the same conditions. The data obtained from the growth assays did not reveal any changes in the growth of Δ*Mopep4* strains grown on CM, MM and MM‐N media, while a weak reduction in vegetative growth of the mutants was observed when cultured on CMII medium, compared to the wild type strain (**Figure** **8**A,B). Moreover, no significant difference was recorded in the amount of conidia produced by the Δ*Mopep4* mutant compared to the Guy11 strain (Figure 8C). Additionally, we conducted pathogenicity assays by inoculating conidial suspensions of both the wild type and the mutant strains onto intact and injured barley rice leaves. Results of the pathogenicity assays showed that the absence of MoPep4 does not affect the virulence of the rice blast fungus on both intact and injured leaf tissues (Figure 8D).

In *S. cerevisiae*, autophagy‐related stress response is dependent on the activities of Pep4 and Prb1 proteases in the vacuole.^[^ ^35^ ^]^ Therefore, we determined whether MoPep4 is also involved in autophagy in *M. oryzae* by analyzing the localization pattern of the autophagosome marker GFP‐MoAtg8 in Guy11 and Δ*Mopep4* mutant strains. The GFP‐MoAtg8 signal was evident in the vacuoles of both Guy11 and Δ*Mopep4* strains even when the fungal hyphae were shifted to nitrogen starvation media containing 2 mM PMSF and grown for an additional 6 h, indicating the normal operation of autophagy in both strains (Figure 8E). Consistently, Western blot analysis revealed that the abundance of GFP gradually increased with increasing time of exposure to nitrogen starvation with corresponding decrease in the abundance of GFP‐MoAtg8 in both strains (Figure 8F). Quantification of the GFP/(GFP+GFP‐MoAtg8) ratio at 0, 2, 4 and 6 h after nitrogen starvation treatment showed that the ratios increased to 0.88 and 0.92, respectively, with no significant difference observed between the two strains (Figure 8G). Taken together, these results suggest that MoPep4 is dispensable for vegetative growth, asexual development, pathogenicity and MoAtg8‐related autophagy in *M. oryzae*.

### 2.10 Targeted Deletion of *MoPEP4* Impacts Pexophagy Process in *M. oryzae*

**Figure 9:**
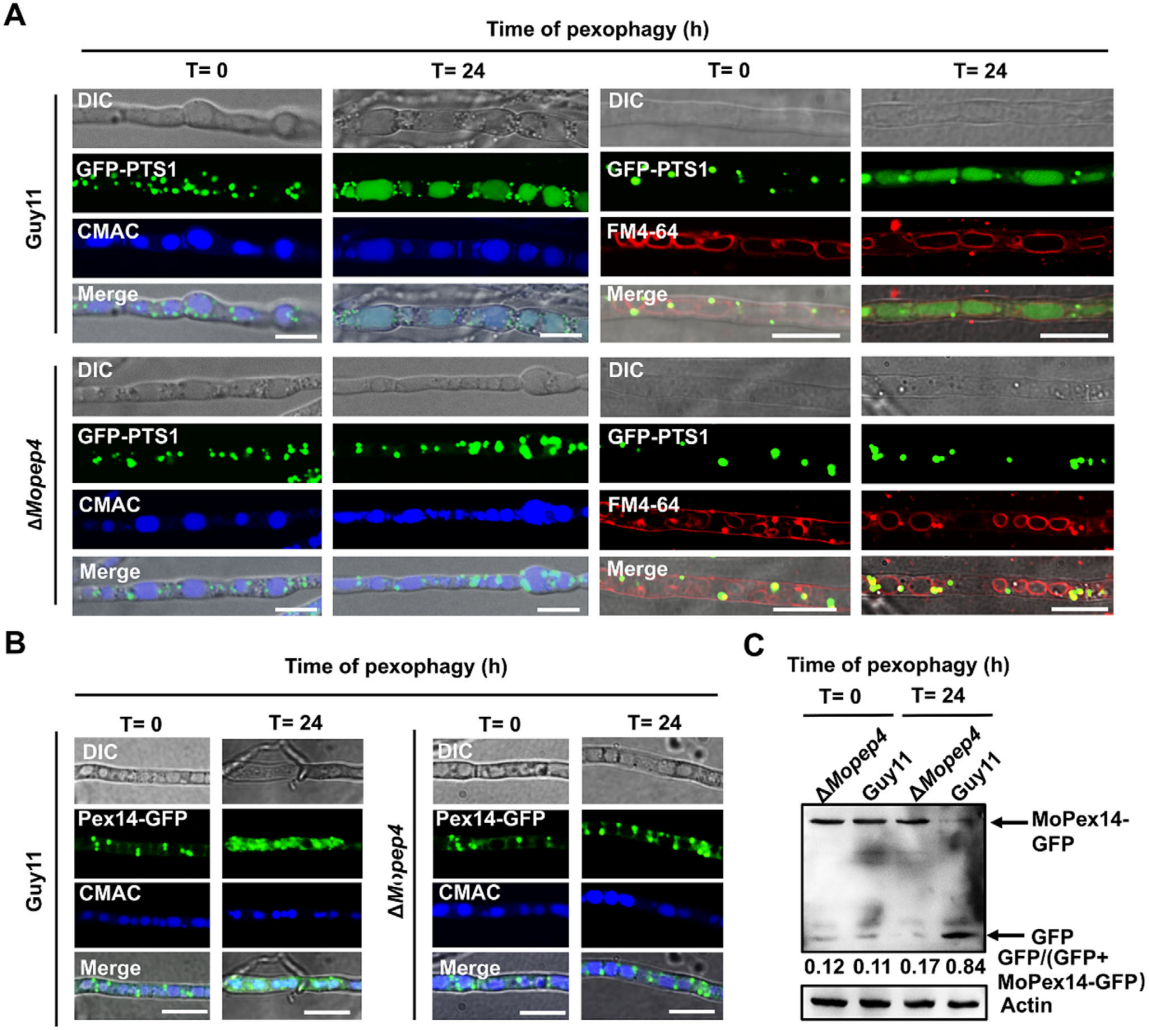
Effects of *MoPEP4* deletions on peroxisome degradation (pexopagy). A,B) Peroxisomes were labeled with the matrix protein GFP‐PTS1 and MoPex14‐GFP, which are peroxisome marker. Prior to the induction of pexophagy (T = 0 h), the peroxisomes were visible as green cytosolic dots and the vacuolar lumen did not display any signals. After induction of pexophagy (T = 24 h), some peroxisomal signals remained in the cytosol, while the vacuoles of Guy11 were filled with diffuse green fluorescence signals, indicating peroxisomal breakdown. However, the Δ*Mopep4* mutant vacuoles lack the GFP‐PTS1 and MoPex14‐GFP fluorescence signals, indicating a defect in pexophagy. The vacuoles and membrane were stained with CMAC and FM4‐64. Bar = 10 µm. C) Δ*Mopep4* mutant showed reduced autophagy flux compared to Guy11. After peroxisome autophagy induction, Guy11 and the Δ*Mopep4* mutant mycelia were collected at each specified time point, total proteins were extracted, and Western blot detection was performed with anti‐GFP. Actin was used as an internal control. Band intensities of the GFP and MoPex14‐GFP were analyzed by ImageJ software (https://imagej.nih.gov/ij/).

In yeast, the aspartyl‐protease Pep4 is known to be required for pexophagy.^[^ ^57^, ^58^ ^]^ To verify the involvement of MoPep4 in pexophagy in *M. oryzae*, an epifluorescent marker of peroxisomes, GFP‐PTS1, was introduced into the protoplast of Guy11, Δ*Moprb1* and Δ*Mopep4* strains, respectively. A pexophagy assay was performed as described in the method, and the hyphae obtained from this assay were examined by confocal microscopy. The results showed that GFP‐PTS1 primarily accumulates in peroxisomes in all strains when the hyphae were grown in nutrient‐rich media for 24 h and then shifted to liquid MM medium containing 2 g L^−1^ Tween 80 for another 24 h to induce peroxisomal proliferation (**Figures** **9**A and S16, Supporting Information). However, upon induction of pexophagy by shifting the hyphae to nitrogen starvation medium and culturing them for an additional 24 h, GFP‐PTS1 diffused into the vacuole lumen of Guy11 and Δ*Moprb1* strains while still remained accumulated in the peroxisomes of Δ*Mopep4* strains (Figures 9A and S16, Supporting Information). Furthermore, we analyzed the degradation of the pexophagy marker protein MoPex14‐GFP in the Δ*Mopep4* strains. The results showed that compared to Guy11, the level of pexophagy was significantly reduced in the Δ*Mopep4/*MoPex14‐GFP strain. After 24 h of induction, the MoPex14‐GFP fluorescence signal in the Δ*Mopep4* mutant remained localized near the vacuolar membrane without entering the vacuole for degradation (Figure 9B). Results from Western blot analysis are consistent with the previous observations that the abundance of free GFP increased with prolonged pexophagy induction time in Guy11 but not in the Δ*Mopep4* mutant, indicating a defect in pexophagy (Figure 9C). Overall, we infer that the deletion of *MoPEP4* gene, but not *MoPRB1* gene, affects the pexophagy process in *M. oryzae*, suggesting that MoPep4 and MoPrb1 have distinct roles in regulating autophagy processes in the rice blast fungus.

### 2.11 Double Deletion of *MoPRB1* and *MoPEP4* Adversely Impacts Asexual Development of *M. oryzae*

**Figure 10:**
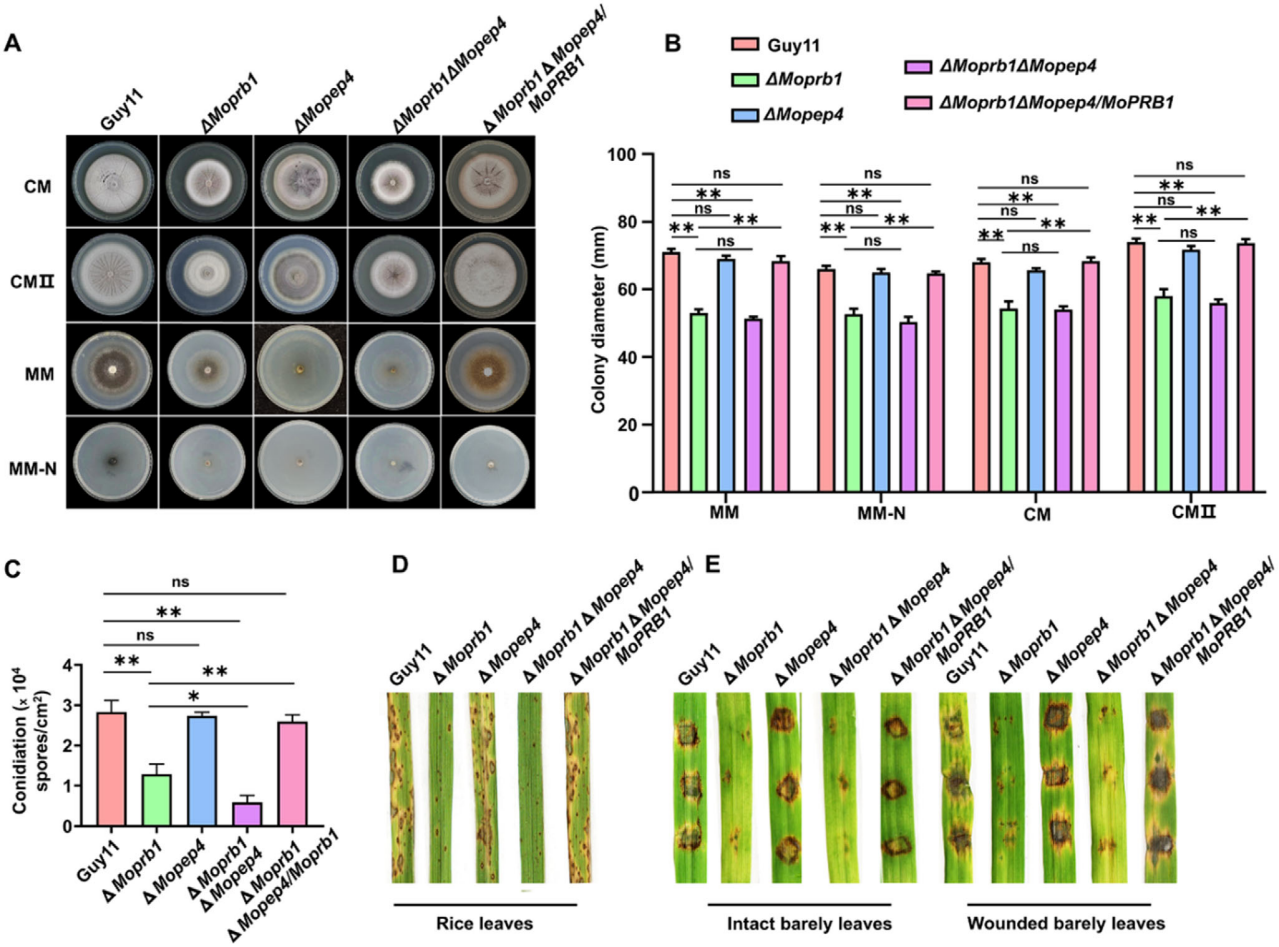
Double deletion of *MoPRB1* and *MoPEP4* affects the mycelial growth, conidiation, and pathogenicity of *M. oryzae*. A) Colony diameters and colony morphologies of the indicated strains after inoculation on CM, CM II, MM, and MM‐N media and subsequent incubation for 7 days at 26 °C. B) Analysis of colony diameters of the indicated strains grown on the different media. Values are presented as mean ± SD from three independent replicates. ^ns^ *p* > 0.05, **p < 0.01. C) Analysis of conidia production by the indicated strains. Values are presented as mean ± SD from three independent replicates, ^ns^ *p *> 0.05, **p < 0.01. D,E) Pathogenicity assays on intact and wounded barley leaves and rice seedlings. The barley leaves were infected with the fungal mycelial blocks while the rice seedlings were infected with conidia suspensions (1 × 10^5^ conidia mL^−1^) obtained from the indicated strains.

Our earlier results showed that MoPrb1 directly interacts with MoPep4. Therefore, we generated a Δ*Moprb1*Δ*Mopep4* double mutant to assess whether there is redundancy in the biological functions of MoPrb1 and MoPep4 in mediating the asexual development and pathogenicity of the rice blast fungus. A decrease in the vegetative growth of Δ*Moprb1*Δ*Mopep4* strains was observed compared to the wild type Guy11 and Δ*Mopep4* when cultured on different types of media, while no significant difference was recorded when compared with Δ*Moprb1* (**Figure** **10**A,B). Conidiation assay revealed a significant reduction in the quantity of spores produced by Δ*Moprb1*Δ*Mopep4* mutant compared to Guy11 and Δ*Mopep4*, and a slight decrease compared to Δ*Moprb1* cultured on RBA (Figure 10C). Pathogenicity tests showed that the combined absence of *MoPRB1* and *MoPEP4* genes impacted the virulence of the rice blast fungus compared to Guy11 and Δ*Mopep4*. However, there was no significant decrease in virulence on intact/wounded barley and rice leaves when compared to Δ*Moprb1* mutant (Figure 10D,E). Additionally, we complemented *MoPRB1* in the Δ*Moprb1*Δ*Mopep4* mutant and observed that the growth, sporulation and pathogenicity of the Δ*Moprb1*Δ*Mopep4/MoPRB1* strain were comparable to those of the wild type Guy11. Altogether, these results suggest that simultaneous loss of *MoPRB1* and *MoPEP4* gene functions exerts significant detrimental effects on the asexual development of *M. oryzae*.

## 3 Discussion

**Figure 11:**
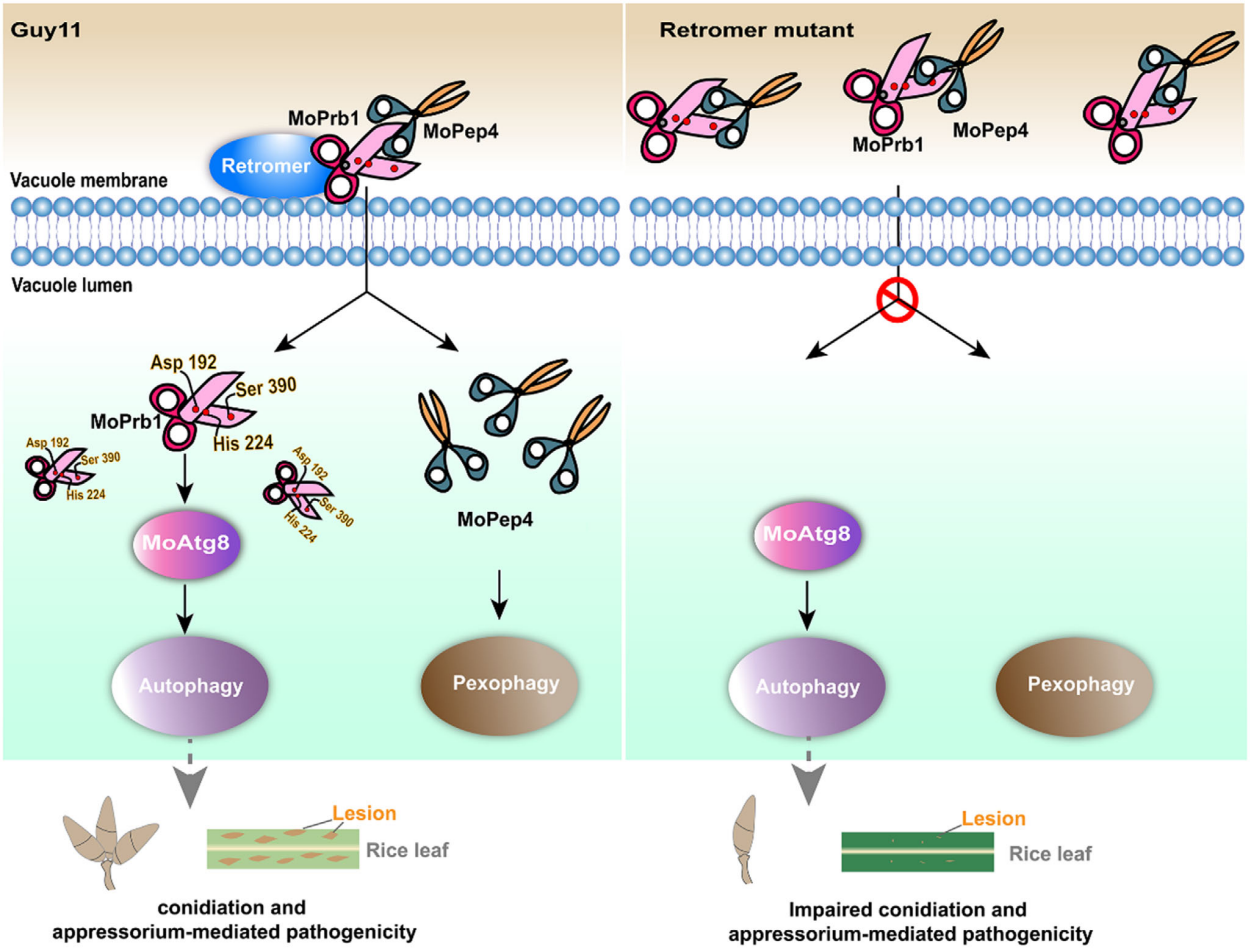
Working model for the functional relationships among the retromer complex, MoPrb1 and MoPep4 in autophagy‐ and pexophagy‐mediated asexual developments and pathogenicity of *M. oryzae*. In the wild type (Guy11), MoPrb1 and MoPep4 are delivered into the vacuole lumen through retromer complex‐dependent orchestration. The key catalytic residues (Asp192, His224, and Ser390) and correct localization of MoPrb1 are critical for regulating the degradation process and MoAtg8‐mediated autophagy, which are essential for vegetative growth, conidiogenesis, and pathogenicity of the rice blast fungus. Meanwhile, MoPep4 is indispensable for the pexophagy process in *M. oryzae*. However, in retromer mutants, MoPrb1 and MoPep4 are unable to enter the vacuole lumen, leading to the mis‐functioning of the degradation pathway. This deficiency ultimately impaired the conidiation and appressorium‐mediated pathogenicity of *M. oryzae*.

Vacuoles, which contain the majority of cellular proteases, are believed to be functionally similar to lysosomes in animals and are responsible for protein turnover.^[^ ^53^ ^]^ In this study, we addressed the question of how the retromer complex is involved in the degradation of autophagic substrates through both macro‐autophagy and micro‐autophagy (**Figure** **11**). We found that the complex regulates both macro‐autophagy and micro‐autophagy by mediating the transport of various vacuolar hydrolases (Figure 11). The key catalytic residues (Asp192, His224 and Ser390) of MoPrb1 and its localization are critical for regulating the degradation process and MoAtg8‐mediated autophagy, which are essential for the vegetative growth, conidiogenesis and pathogenicity of the rice blast fungus (Figure 11). Furthermore, the retromer complex recruits the vacuolar aspartyl protease MoPep4 via MoPrb1 to mediate pexophagy. However, this pathway is not required for the fungal development or virulence (Figure 11). Although MoPrb1 and MoPep4 are able to interact in retromer null mutants, they are unable to be delivered to the vacuole lumen, leading to perturbation of the degradation process (Figure 11). Overall, our results provide clear evidence that the retromer complex targets a specific vacuolar protease to the vacuole lumen to ensure degradation of autophagic substrates for *M. oryzae* development and plant infection.

It was previously reported that the retromer complex and Prb1‐Pep4 are associated with the autophagy process in fungi and mammals.^[^ ^45^, ^58^, ^59^ ^]^ However, the direct connections between the vesicle trafficking machinery (the retromer complex) and the vacuolar proteases Prb1 and Pep4 have not been established. We used high throughput proteomics to screen interacting proteins of MoVps35, which is the core component of the retromer cargo selective complex. Both MoPrb1 and MoPep4 were captured in the assay. Compared to MoPep4, MoPrb1 was captured three times, indicating that the interaction between MoVps35 and MoPrb1 is stronger and more stable. We used Y2H, BiFC and colocalization analysis to further confirm the physical interaction between MoVps35 and MoPrb1. All of these approaches showed positive interaction between MoVps35 and MoPrb1. Interestingly, our BiFC and colocalization results further showed that the interaction between MoVps35 and MoPrb1 occurs on some punctate structures near the vacuolar membrane, possibly on endosomes and/or the trans‐Golgi network (TGN), which are both retromer‐localized compartments.^[^ ^25^, ^29^ ^]^ In yeast, both Prb1 and Pep4 are transported to the vacuole in zymogen form and activated in a complex cascade.^[^ ^54^, ^60^ ^]^ Typically, the zymogen preproPrb1 undergoes autocatalytic cleavage in the ER, removing a 260‐amino‐acid propeptide from the N‐terminus.^[^ ^61^ ^]^ Following that, the proPrb1 is further modified with O‐linked glycans in the Golgi.^[^ ^49^ ^]^ With this information, we hypothesize that MoVps35 may recognize immature MoPrb1.

In yeast, Pep4 and Prb1 are both localized to the vacuole where they function cooperatively to activate a range of vacuolar hydrolases. Pep4 undergoes autocatalytic processing to become activated, whereas the maturation of Prb1 relies on cleavage by Pep4.^[^ ^32^, ^62^ ^]^ Interestingly, our findings reveal an asymmetric regulatory relationship between MoPrb1 and MoPep4. We observed that the correct vacuolar localization of MoPep4 is dependent on MoPrb1, whereas the localization of MoPrb1 is not affected by the deletion of *MoPEP4*. Western blot analysis further showed that the degradation of MoPrb1‐GFP was not significantly altered in the Δ*Mopep4* mutant, while GFP‐MoPep4 could still be detected in the Δ*Moprb1* mutant. These results suggest that MoPrb1 activation may not solely depend on MoPep4 in *M. oryzae*, suggesting that additional proteases may participate in MoPrb1 maturation or activation. In contrast, the activation of MoPep4 appears to be tightly regulated by MoPrb1, highlighting a unidirectional dependency. Moreover, another interesting observation was that the retromer complex is responsible for targeting MoPrb1 and MoPep4 (through MoPrb1) to the lumen of the vacuole. Loss of any retromer component, such as MoVps35, MoVps26, MoVps29 or MoVps17, leads to mislocalization of MoPrb1‐GFP and GFP‐MoPep4 to the outer region of the vacuole in dot‐like structures. Careful observation showed that many of these punctate structures are close to the vacuolar membrane. In yeast, the retromer cargo that has been studied the most is Vps10.^[^ ^26^, ^63^ ^]^ Vps10 is a single‐pass type‐I transmembrane protein and is found in punctate locations, on endosomes and the Golgi.^[^ ^63^ ^]^ In the absence of any retromer subunit, Vps10 accumulates on the vacuolar membrane and fails to recycle back from the endosome to the Golgi.^[^ ^63^ ^]^ In our previous study, we found that MoVps10 localizes to punctate structures and colocalizes with MoVps17 in *M. oryzae*.^[^ ^29^ ^]^ However, MoVps10 is not necessary for the development and pathogenicity of the rice blast fungus, suggesting that other unknown interacting proteins might be responsible for the defects observed in the retromer mutants.^[^ ^29^ ^]^ In summary, MoPrb1 is considered as a new interacting protein of the retromer complex and this interacting protein is essential for MoPep4 functions. We were also curious as to why the loss of retromer components prevents MoPrb1‐GFP and GFP‐MoPep4 from entering the vacuole lumen. We coined several hypotheses regarding this phenomenon. One is that unknown receptors that bind to MoPrb1 and MoPep4 are mis‐localized when any of the retromer subunits is lacking. Another possibility is that there could be an alteration in the pH of the vacuole which possibly rendered MoPrb1 and MoPep4 functionless. A third hypothesis is that the activation of the zymogen cascade might be impaired in retromer null mutants. Further research should analyze these aspects to unveil the underlying molecular mechanisms.

In yeast, Prb1 is a serine endoprotease belonging to the S8 family, whereas Pep4 is an aspartyl endoprotease belonging to the A1 family of proteases.^[^ ^53^, ^54^ ^]^ In this study, we observed that the MoPrb1 and MoPep4 proteins exhibited significant sequence similarity to the Prb1 and Pep4 proteins of yeast, respectively, with a similarity of over 54%. Interestingly, despite the conservation of MoPrb1 and MoPep4 sequences with respect to their yeast orthologs, these two proteins perform different functions in the rice blast fungus. MoPrb1 regulates macro‐autophagy and is crucial for conidiation and pathogenicity in *M. oryzae*. On the other hand, MoPep4 regulates pexophagy but is dispensable for asexual development and virulence in *M. oryzae*. Additionally, previous research has demonstrated the existence of nonselective and selective autophagy in *M. oryzae*, each with distinct roles in fungal morphogenesis and pathogenesis.^[^ ^8^, ^12^ ^]^ Moreover, our results showed that although the transport of MoPep4 is regulated by MoPrb1, the Δ*Moprb1* mutant does not participate in pexophagy. This suggests that multiple compensatory pathways for pexophagy may exist in *M. oryzae* and that this process may be regulated by different vacuolar proteases. The disruption of one protease‐mediated pexophagy pathway might activate alternative mechanisms to compensate for its loss, although the exact mechanisms remain to be further elucidated. Our findings are however in contrast to those reported in *U. maydis* ^[^ ^40^ ^]^ where Pep4 was shown to be essential for host invasion. This functional contradiction in *M. oryzae* (a hemibiotrophic ascomycete) and *U. maydis* (a biotrophic basidiomycete) could be attributed to differences in nutrient requirement/content and distinct plant interaction strategies. Overall, this study provides the first comprehensive analysis of the role of the retromer complex in regulating macro‐autophagy and micro‐autophagy through two distinct vacuolar endoproteases, thus highlighting their biological relevance in the rice blast pathogen.

## 4 Experimental Section

### Affinity Purification and Mass Spectrometric Analysis

For affinity purification, fresh mycelia from MoVps35‐GFP were harvested in liquid CM and ground into fine powder in liquid nitrogen. Subsequently, the samples were lysed in extraction buffer (10 mm Tris HCl pH 7.5, 150 mm NaCl, 0.5 mM EDTA, 1% Triton X‐100, 2 mm PMSF) containing protease inhibitor cocktail (Cat. no. C510026, Sangon Biotech, Shanghai, China) for 30 min. The samples were centrifuged at 12 000 rpm for 20 min at 4 °C, and the total cell lysates were incubated with 30 µL GFPTrap _A beads (Cat. no. gta‐20, ChromoTek Inc., Planegg‐Martinsried, Germany) at 4 °C for 4 h. Finally, the bound proteins were eluted by heating at 100 °C for 10 min. Mass spectrometry analysis was performed as previously reported.^[^ ^42^ ^]^

### Construction and Screening of *M. oryzae* cDNA Library

To identify MoVps35‐interacting proteins, Y2H screen was performed using the Matchmaker GAL4 Yeast Two‐Hybrid System (Clontech, USA), according to the manufacturer's manual. RNA was extracted from Guy11 mycelia that were pre‐cultured in an autophagy‐induced condition. Total RNA was extracted from the samples and used for Y2H library construction. cDNA library was constructed by OE Biotech (Shanghai, China) using the CloneMiner II cDNA Library Construction Kit (Cat. no. A11180, Invitrogen). Before screening the *M. oryzae* cDNA library, the pGBKT7‐MoVps35 vector was constructed and a self‐activation verification assay was performed. The results showed that pGBKT7‐MoVps35 had no self‐activating activity in the Y2H Gold strain. Subsequently, the pGBKT7‐MoVps35 vector and *M. oryzae* cDNA library were co‐transformed into the Y2H Gold strain, followed by inoculation on SD/‐Ade/‐His/‐Leu/‐Trp/X‐α‐Gal/AbA medium. There were 48 clones that turned blue after color development. The blue colonies were isolated and sequenced. Finally, 39 different genes were identified (Table S1, Supporting Information).

### Strains and Culture Conditions

The *M. oryzae* isolate Guy11 was used as the wild‐type (WT) strain throughout this study. Gene deletion mutants were genetically derived from Guy11. All strains were stored on dried filter paper stocks at −20 °C and revived on complete medium (CM, 6 g L^−1^ yeast extract, 6 g L^−1^ casein hydrolysate, 10 g L^−1^ sucrose, 20 g L^−1^ agar) at 26 °C as described previously.^[^ ^64^ ^]^ For vegetative growth, the various strains were cultured on CM, complete medium II (CMII, 10 g L^−1^ D‐glucose, 2 g L^−1^ peptone, 1 g L^−1^ yeast extract, 1 g L^−1^ casamino acid, 6 g L^−1^ NaNO_3_, 1.52 g L^−1^ KH_2_PO_4_, 0.52 g L^−1^ KCl, 0.5 g L^−1^ MgSO_4_·7H_2_O, 1 mL L^−1^ vitamin solution, 1 mL L^−1^ trace elements, and 20 g L^−1^ agar), minimal medium (MM, 10 g L^−1^ D‐glucose, 6 g L^−1^ NaNO_3_, 1.52 g L^−1^ KH_2_PO_4_, 0.52 g L^−1^ KCl, 0.5 g L^−1^ MgSO_4_·7H_2_O, 0001% thiamine, 1 mL L^−1^ trace elements, and 20 g L^−1^ agar), and minimal medium without nitrogen (MM‐N, also referred to as nitrogen starvation media, 10 g L^−1^ D‐glucose, 1.52 g L^−1^ KH_2_PO_4_, 0.52 g L^−1^ KCl, 0.5 g L^−1^ MgSO_4_·7H_2_O, 0001% thiamine, 1 mL L^−1^ trace elements, and 20 g L^−1^ agar) at 26 °C for 7 days. For conidiation, the individual strains were cultured on rice bran agar (RBA) medium (40 g L^−1^ rice bran, 20 g L^−1^ agar, and pH 6.0) in a light‐free environment at 26 °C for 7 days, followed by transfer to an incubator with continuous illumination for 3 days after removing the vegetative hyphae. All gene deletion mutants, complemented strains, and fusion‐expression strains in this study are listed in Table S2 (Supporting Information).

### Targeted Gene Deletion and Complementation

The “split‐marker” gene deletion strategy was utilized for targeted gene deletion of *MoPRB1* and *MoPEP4* genes, as shown in Figures S3A and S15A (Supporting Information). The primer pairs MoPRB1‐AF/AR and MoPEP4‐AF/AR were employed to amplify the upstream flanking fragments of the open reading frames (ORFs) of *MoPRB1* and *MoPEP4*, while the primer pairs MoPRB1‐BF/BR and MoPEP4‐BF/BR were utilized for amplifying the downstream flanking fragments. Subsequently, two flanking fragments of each gene were respectively ligated with overlapping fragments of the hygromycin B phosphotransferase gene (*HPH*) amplified using primer pairs HYG‐F/HY‐R and YG‐F/ HYG‐R by overlap extension PCR. The PCR products of each gene were co‐transformed into protoplasts of the wild‐type strain (Guy11). The transformants were initially screened using PCR with primer pairs OF/OR and UA/H853. Successful knockout candidates were further validated and confirmed through Southern blot analysis. To perform complementation and subcellular localization analysis, a 2.60‐kb fragment containing the native promoter and ORF of *MoPRB1* and a 3.44‐kb fragment containing the native promoter and ORF of *MoPEP4* were respectively amplified and then inserted into the vector pKNTG, which contains neomycin‐resistance gene and GFP gene in‐frame (Table S3, Supporting Information). Afterward, the constructed vector was reintroduced into the protoplasts of the respective mutant strains. Transformants were screened for neomycin resistance, and confirmed by PCR using the primer pair MoPRB1‐CF/GFP‐R or GFP‐PEP4‐F/ GFP‐PEP4‐R. The oligonucleotide primers used are provided in Table S4 (Supporting Information).

### Yeast Two‐Hybrid Assays

The full‐length cDNA of the target gene was amplified from Guy11 and cloned into the prey vector pGADT7 and/or the bait vector pGBKT7. The constructs were co‐transformed into the yeast competent cell AH109. All transformants were grown on SD‐Leu‐Trp and SD‐Leu‐Trp‐His‐Ade media supplemented with 20 mg mL^−1^ X‐α‐gal at 30 °C for 5 days. Interaction of pGADT7‐T and pGBKT7‐53 was used as positive control while that of pGADT7‐T and pGBKT7‐Lam (BD‐Lam) was used as negative control. The plasmids of bait and prey vectors are described in Table S3 (Supporting Information). The corresponding oligonucleotide primers used are provided in Table S4 (Supporting Information).

### Bimolecular Fluorescence Complementation (BiFC) Assays

BiFC assays were performed to identify the interaction relationship between MoVps35 and MoPrb1 in vivo as previously described.^[^ ^65^ ^]^ The coding sequences of yellow fluorescence protein (YFP) were split into two halves and respectively fused at the carboxyl termini of MoVps35, MoPrb1, and MoPep4 to obtain the MoVps35‐cYFP, MoVps35‐nYFP, MoPrb1‐nYFP, and cYFP‐MoPep4 constructs under the control of native gene promoter. To verify the protein interactions among MoVps35, MoPrb1, and MoPep4, different BiFC assays were designed. In Guy11 protoplasts, the interaction between MoVps35 and MoPrb1 was tested by co‐transforming MoVps35‐cYFP and MoPrb1‐nYFP, while the interaction between MoVps35 and MoPep4 was examined by co‐transforming MoVps35‐nYFP and cYFP‐MoPep4. Similarly, the interaction between MoPrb1 and MoPep4 was assessed by co‐transforming MoPrb1‐nYFP and cYFP‐MoPep4. Additionally, to investigate the interaction between MoVps35 and MoPep4 in the Δ*Moprb1* mutant, and the interaction between MoPep4 and MoPrb1 in ΔMovps35, MoVps35‐nYFP, and cYFP‐MoPep4 were co‐transformed into the protoplasts of the Δ*Moprb1* mutant. MoPrb1‐nYFP and cYFP‐MoPep4 were co‐transformed into the protoplast of the Δ*Movps35* mutant Also, co‐transformation of MoVps35‐cYFP vector with nYFP vector and cYFP vector with MoPrb1‐nYFP vector, cYFP‐MoPep4 vector with nYFP vector, and cYFP vector with MoVps35‐nYFP vector into Guy11 protoplasts served as negative controls. Similarly, the cYFP‐MoPep4 vector with nYFP vector and cYFP vector with MoVps35‐nYFP vector into the Δ*Moprb1* mutant protoplasts also served as negative controls. Transformants were initially screened by both hygromycin and neomycin resistance, and potential candidates were further validated by PCR (Table S4, Supporting Information). Live‐cell imaging was performed to observe the reconstituted fluorescence signals using a Nikon A1 laser confocal microscope (Nikon, Japan).

### Conidiogenesis, Conidial Germination, Appressorium Development, and Turgor Pressure Assays

Conidiation, conidial germination, and appressorial formation ability of *M. oryzae* were evaluated following previously established protocols with minor modifications.^[^ ^66^ ^]^ Briefly, conidia harvested from RBA medium were counted and divided by the corresponding colony areas to determine conidia density per unit area. To observe conidiophore and conidial development, blocks of fully colonized RBA medium were cut and placed on micro‐slides. The slides were then incubated under light for 3 days and observed under a light microscope. For assessing conidia germination and appressoria formation, a 20 µL conidial suspension (adjusted to a concentration of 1 × 10^5^ conidia mL^−1^) harvested from RBA medium was pipetted and dropped onto hydrophobic coverslips and incubated in a dark, humid chamber at 26 °C for 2, 4, 8, 12, and 24 h. The rate of conidia germination and appressoria formation was observed and recorded using microscopic examination. Appressorium turgor was measured by incipient cytolysis assay using a 0, 2, and 3 molar concentration of glycerol solution, as previously described.^[^ ^67^ ^]^ At least 100 conidia were counted per biological replication, and the experiment was performed in triplicate.

### Pathogenicity Assays

For barley leaf infection experiments, the various strains were cultured on CM media for 5 days. Mycelial blocks of equal size were then cut and inoculated onto intact or wounded 10‐day‐old barley leaves. The inoculated plant leaves were initially incubated in a dark chamber at 25 °C with a stable relative humidity of 90% for 24 h. Subsequently, the leaves were transferred to a chamber with a photoperiod of 12/12 h of light/dark cycles. For rice infection assays, conidial suspensions (1 × 10^5^ conidia mL^−1^ in 0.02% Tween 20 solution) were prepared from RBA media and used for spray‐inoculation on 3‐week‐old rice seedlings (*O. sativa* cv. CO39). Disease development and lesion formation were assessed at 7 days post‐inoculation (dpi) for both barley and rice infection assays. Leaf‐sheath inoculation assay was carried out on 5‐week‐old rice leaves, following the procedure described in a previous report.^[^ ^68^ ^]^ The growth of invasive hyphae (IH) in rice cells was observed and recorded under a microscope at 40 h post‐inoculation (hpi). The different types of IH formed were quantified and subjected to statistical analysis.

### Autophagy and Pexophagy Analyses

A GFP‐MoATG8 construct was initially introduced into the protoplasts of both the wild‐type Guy11 and the gene deletion mutants. Strains expressing the GFP‐Atg8 were cultured in liquid CM at 26 °C for 24 h. After thorough washing with sterilized double‐distilled water (ddH_2_O), the mycelia were subjected to nitrogen starvation by culturing them in liquid MM‐N with 2 mm phenylmethylsulfonyl fluoride (PMSF) for 2, 4, and 6 h. Mycelia samples from each time point were stained with 7‐amino‐4‐chloromethylcoumarin (CMAC, Cat. no. HY‐D1462, MedChemExpress) and observed under a Nikon A1 laser confocal microscope (Nikon, Japan). Additionally, mycelia from Guy11 and the gene deletion mutants were collected before and after autophagy induction (at 0, 2, 4, and 6 h) for total protein extraction. Western blot analysis using anti‐GFP antibody (1: 5000, Cat. no. M20004, Abmart, China) was performed to assess the GFP‐MoAtg8 cleavage, with actin serving as the loading control.

Pexophagy assays were conducted as described previously, with minor modifications.^[^ ^69^ ^]^ Strains expressing the epifluorescent marker for peroxisomes, pexophagy GFP‐PTS1 and MoPex14‐GFP^[^ ^70^ ^]^ were initially grown in liquid CM under continuous shaking at 26 °C in darkness for 48 h. Subsequently, to induce the peroxisomal proliferation, the mycelia were transferred to liquid MM containing 2 g L^−1^ Tween 80 at 26 °C for 24 h. Pexophagy was induced by washing the mycelia 3 times with sterilized ddH_2_O (T = 0 h) and then shifting them to liquid MM‐N for an additional 24 h (T = 24 h). Hyphae obtained from this assay were examined using a confocal microscope at the indicated time points (T = 0 h and T = 24 h). For biochemical quantification analysis, mycelial samples expressing MoPex14‐GFP were treated as described above to induce pexophagy. After harvesting the mycelia, total protein was extracted for immunoblot analysis of MoPex14‐GFP.

### Live‐Cell Imaging

To investigate the subcellular localizations of the target proteins during vegetative growth, the fungal strains were initially cultured in liquid CM at 26 °C for 48 h. For localization in conidia and during appressorial development, conidia suspensions from the various strains were dropped onto hydrophobic coverslips (Fisher Scientific) and incubated in a dark and humid chamber at 25 °C for 4 h. To confirm the vacuolar lumen localization of the protein, hyphae from the cultured strains were harvested and stained with 10 µM CMAC and 8 µm FM4‐64 dye (Cat. no. T3166, Invitrogen). Lipid peroxides were detected with C11‐BODIPY581/591 (S0043, Beyotime). Imaging was performed using either an Olympus DP72 fluorescent microscope or a Nikon A1 Plus confocal microscope (Japan). GFP fluorescence signal was visualized at 488 nm wavelength laser excitation, while FM4‐64, mCherry, and mScarlet fluorescence signals were observed at 561 nm wavelength laser excitation. CMAC fluorescence signal was captured at 650 nm wavelength laser excitation.

### Protein Extraction and Western Blot

Hyphae from the respective strains were cultured and harvested from CM liquid media. The hyphal samples were ground into fine powder and ≈1 g of the each of the powdered sample was collected and lysed in a lysis buffer (10 mm Tris HCl pH 7.5, 150 mm NaCl, 0.5 mm EDTA, 1% Triton X‐100, 2 mm PMSF) supplemented with a protease inhibitor cocktail (Cat. no. C510026, Sangon Biotech, Shanghai, China). The solutions were centrifuged at 14000 rpm for 10 min at 4 °C. The resulting supernatants were mixed with 5 × protein loading buffer and boiled at 95 °C for 5 min. The crude protein extracts were subsequently separated by sodium dodecyl sulfate polyacrylamide gel electrophoresis (SDS‐PAGE) and analyzed by Western blot using the corresponding antibodies.

### Statistical Analysis

All experimental data were presented as mean ± standard deviation (SD) from three independent experiments. Statistical significance between groups was evaluated using one‐way ANOVA and *t*‐tests conducted in SPSS 17.0, with *p* < 0.05 indicating significance.

## Author Contributions

W.Z. and Z.W. contributed to conceptualization and resources and funding acquisition. D.Z., J.H., Y.H., Y.F., M.P., S.L., H.Z., and N.J. contributed to methodology and investigation. W.Z., D.Z., J.H., Y.H., L.L., Y.S.A., N.I.N., and X.P. contributed to visualizing and/or analyzing the data. W.Z., Y.H., N.I.N., J.H., and D.Z. contributed to writing the original draft review and/or editing. D.Z., J.H., and Y.H. contributed equally to this work.

## Conflict of Interest

The authors declare no conflict of interest.

## Supporting information



Supporting Information

Supporting Information

## Data Availability

The data that support the findings of this study are available from the corresponding author upon reasonable request.
