# Supplementary material for: Retromer Regulates Macro‐ and Micro‐Autophagy via Distinct Vacuolar Proteases in the Rice Blast Fungus
Source: Adv Sci (Weinh). 2025 Aug 21;12(41):e10068. doi: 10.1002/advs.202510068 (PMC12591164; doi:10.1002/advs.202510068)
Supplement: Supplementary file 1 — Supporting Information [file ADVS-12-e10068-s001.docx]

**Supplementary Figures and Figure legends**


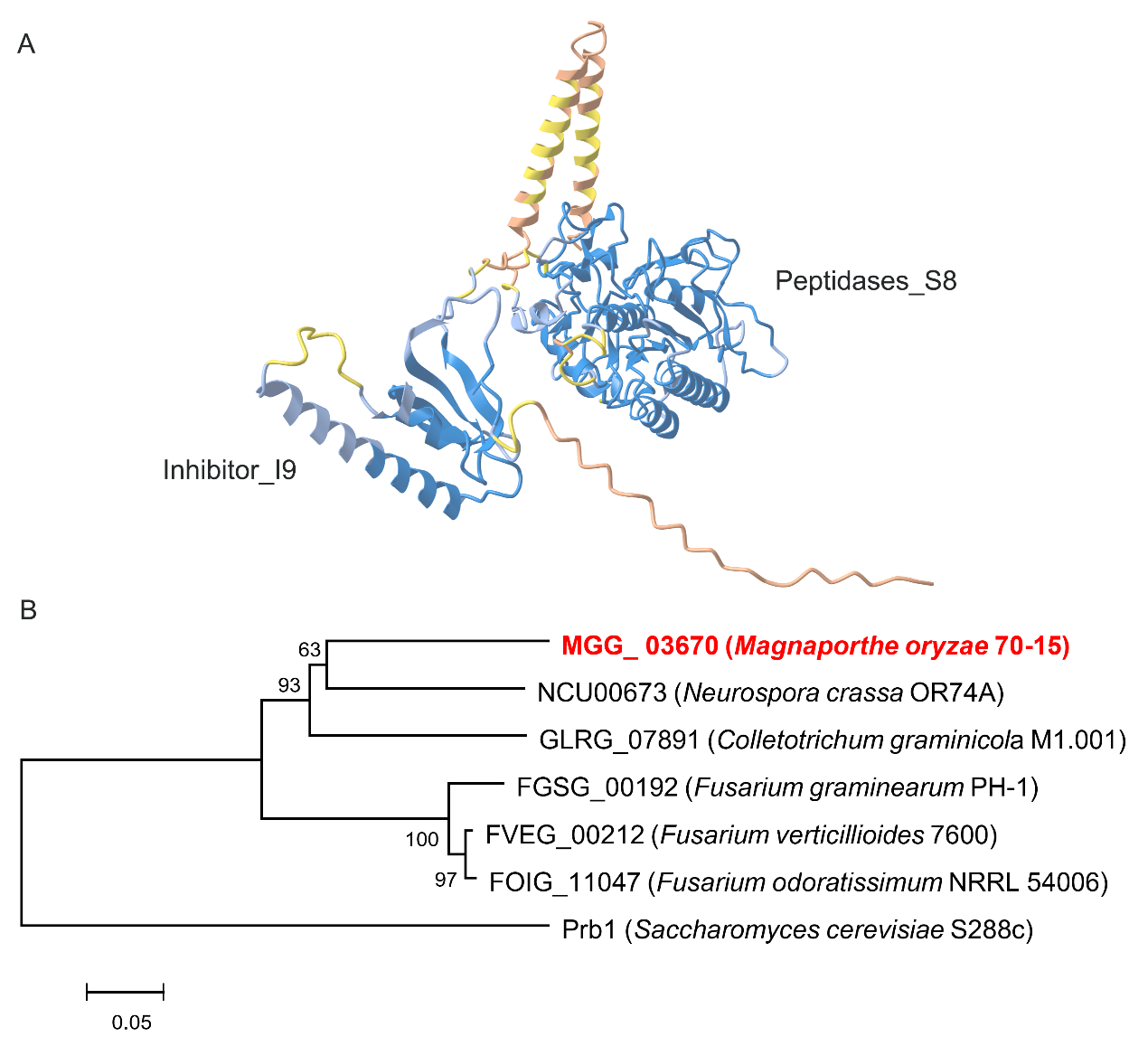


**Fig. S1 Domain and phylogenetic analyses of Prb1 homologs.** (A) 3D structure and domains prediction of MoPrb1 protein. (B) A neighbor-joining tree was constructed based on the amino acid sequences of Prb1 homologs from different species. The numbers at the nodes represent the percentages of their occurrences in 10, 000 bootstrap replicates.


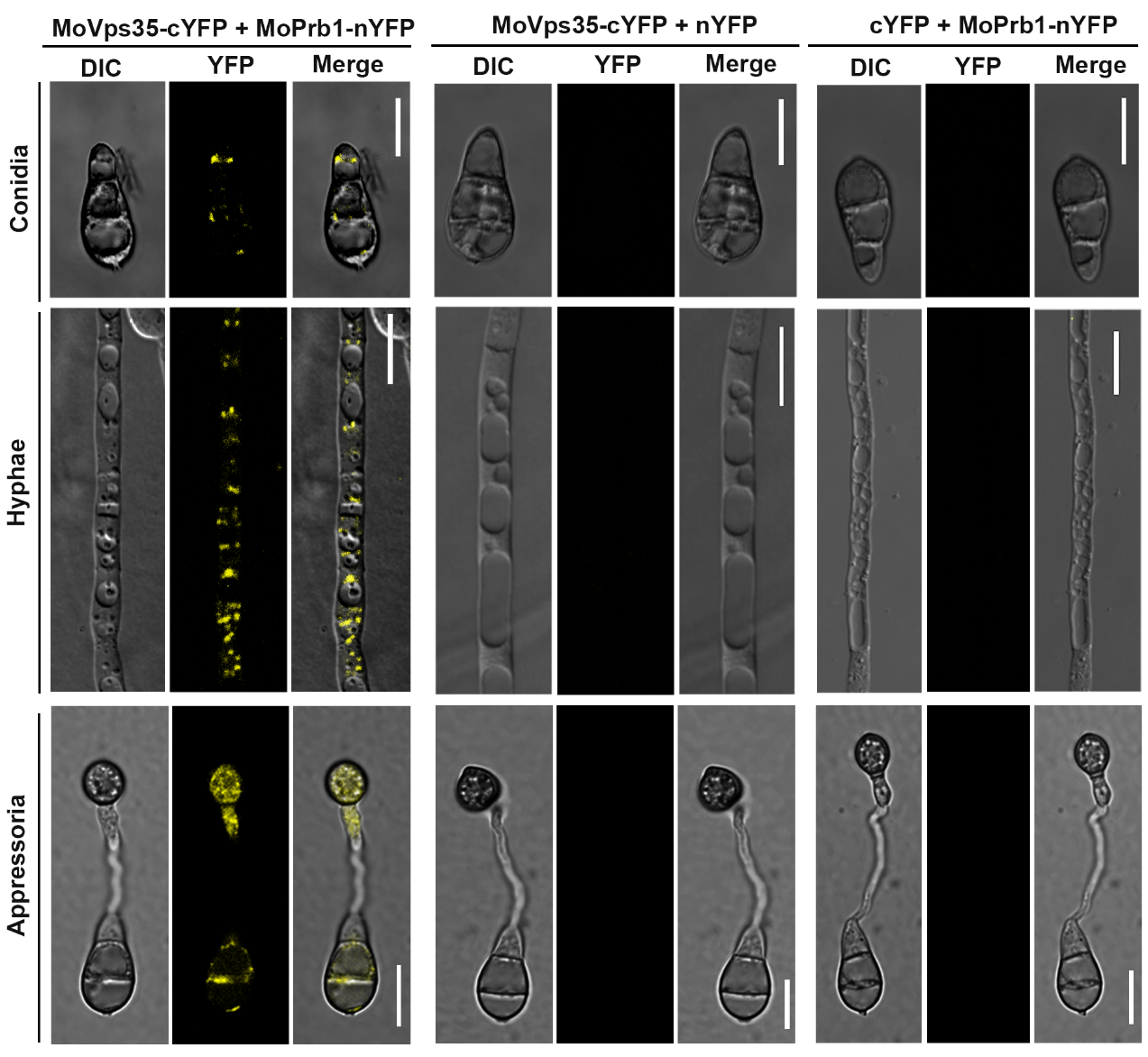


**Fig. S2 Bimolecular fluorescence complementation (BiFC) assay was used to visualize the interactions between MoVps35 and MoPrb1 *in vivo*.** Reconstituted YFP signals were only observed in the strain expressing MoVps35-cYFP and MoPrb1-nYFP constructs. Bar = 10 μm.


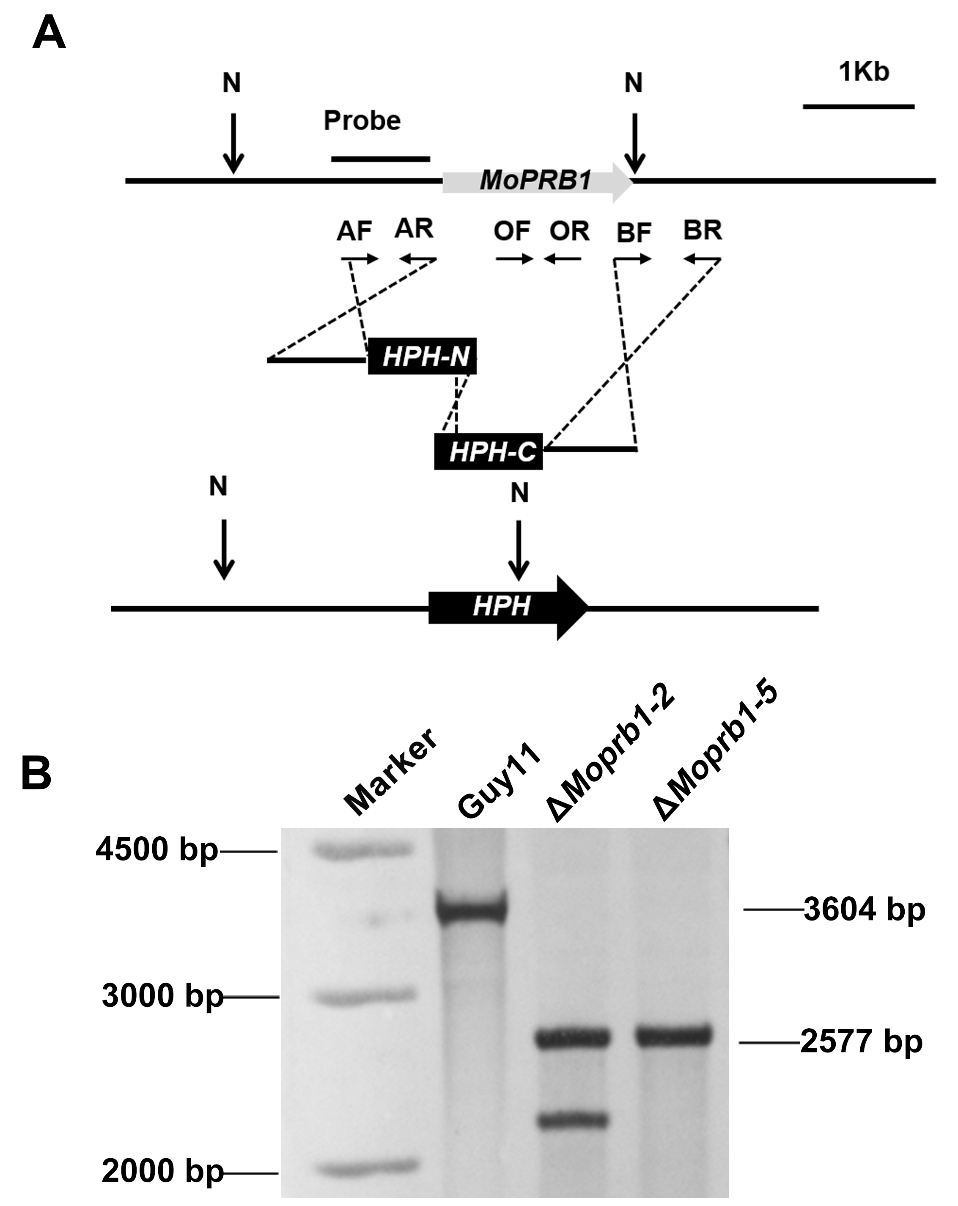


**Fig. S3 Strategy for deletion and confirmation of *MoPRB1* gene in *M. oryzae*.** (A) Gene deletion strategy for *MoPRB1*. (B) Southern blot confirmation of *MoPRB1* gene deletion. *Nde*I was used for genomic DNA digestion and the upstream fragment (amplified by the primer pair AF/AR) was used as a probe. The 3.6 kb target band was only present in Guy11, while the 2.5 kb target band was only detected in the gene deletion mutants as predicted. The Δ*Moprb1-5* transformant was used for subsequent analysis.


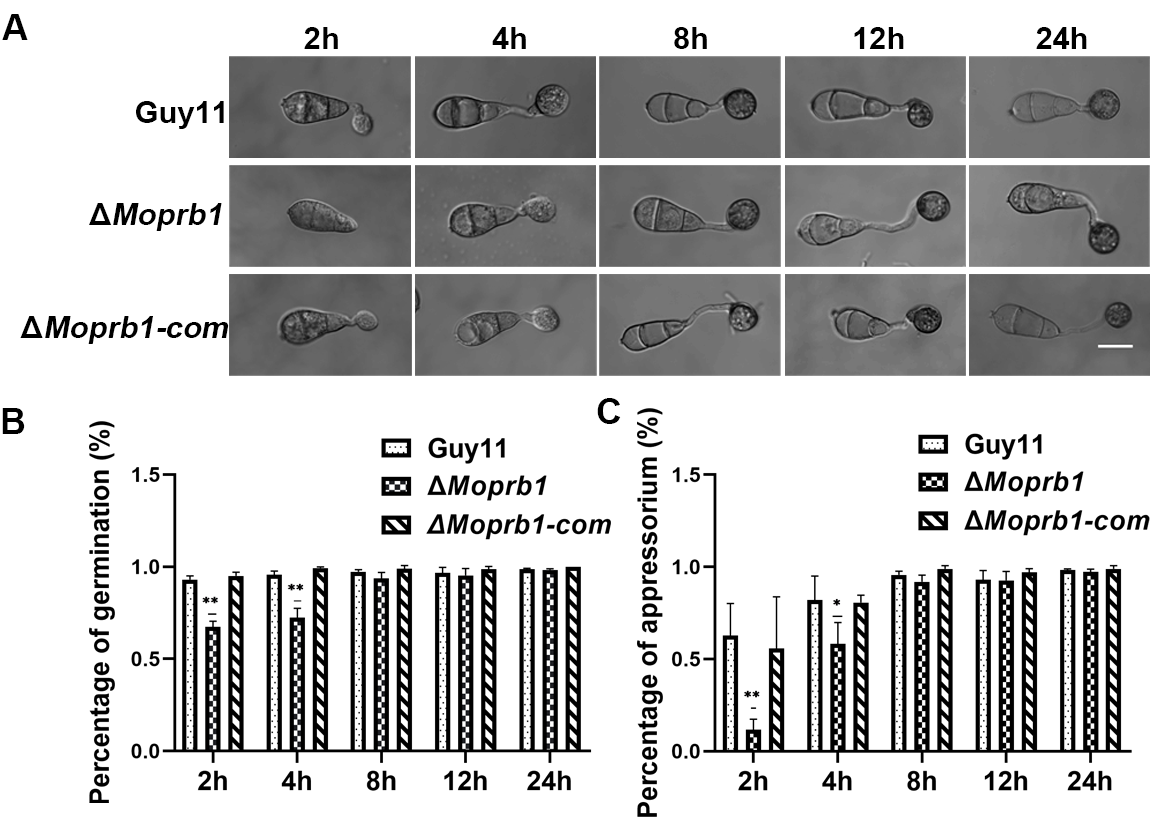


**Fig. S4 Deletion of *MoPRB1* does not significantly affect appressorial development in *M. oryzae*.** (A) Morphologies of Guy11, the *∆Moprb1* mutant and the complemented strain *∆Moprb1-*com during conidial germination and appressorial development. Bar = 10 µm. (B) Percentage of conidial germination for each strain after 2, 4, 8, 12 and 24 hours of incubation. (C) Percentage of appressorial formation by the indicated strains after 2, 4, 8, 12, and 24 hours of incubation. At least 100 conidia were counted per biological replication and the experiment was performed in triplicate. Means and standard deviations were calculated from three independent biological replicates. ^*^, *P* < 0.05; **, *P* < 0.01.


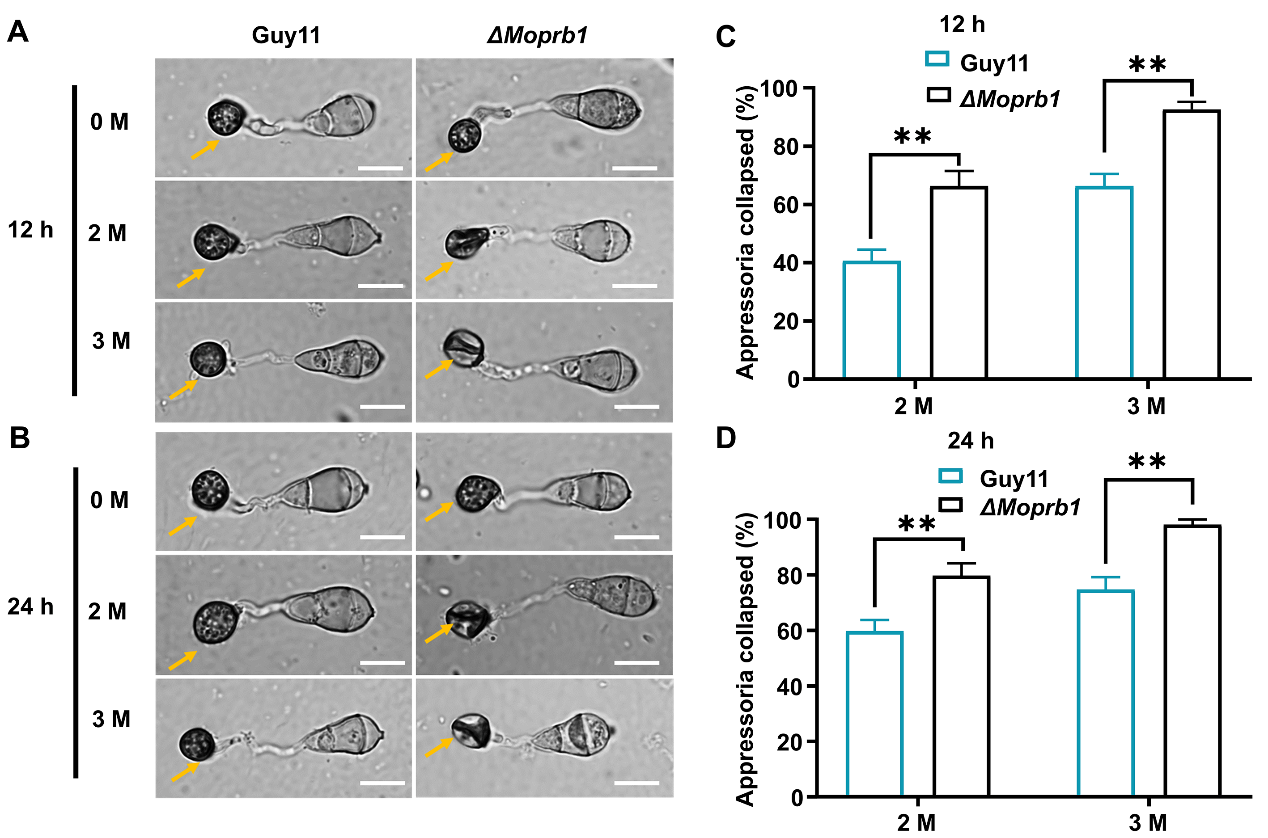


**Fig. S5** **MoPrb1 regulates appressorium turgor generation.** (A, B) Appressorium turgor was measured by incipient cytorrhysis assays. Appressoria were allowed to form on plastic coverslips for 12 h or 24 h, and the collapsed appressoria were assessed after exposure to 2 M or 3 M glycerol solutions. Yellow arrows indicate appressoria formed by Guy11 or the Δ*Moprb1* strain at 12 h or 24 h, followed by exposure to 2 M or 3 M glycerol solution to assess collapse. Bar = 10 μm. (C, D) Proportion of collapsed appressoria after exposure of conidia to 2 M or 3 M glycerol. At least 100 conidia were counted per biological replication and the experiment was performed in triplicate. Means and standard deviations were calculated from three independent biological replicates. **, *P* < 0.01.


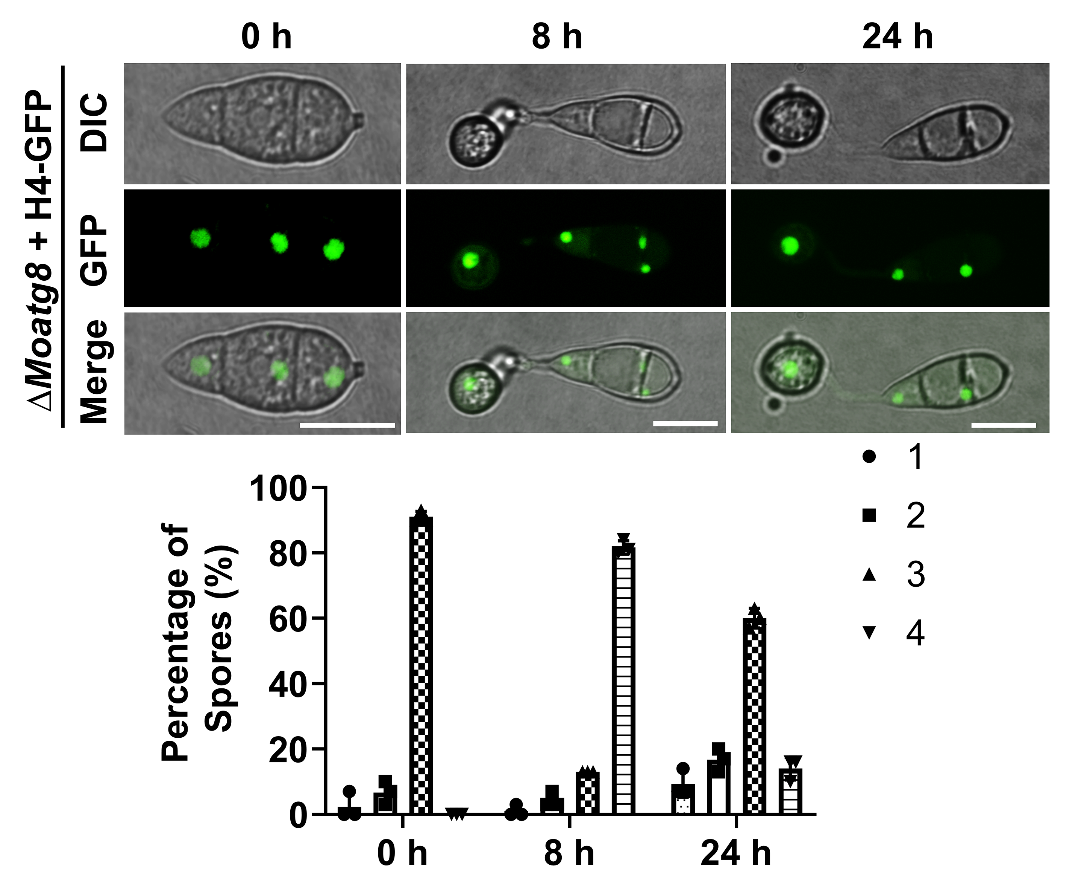


**Fig. S6 The autophagy-deficient mutant strain Δ*Moatg8* delays nuclear degradation in *M. oryzae*.** Confocal microscopy images showed delayed nuclear degradation in Δ*Moatg8* mutant conidia during appressorial development in *M. oryzae*. The bar chart illustrates the percentage of spores containing 0 to 4 nuclei in the Δ*Moatg8* mutant during appressorial development (n = 100, three replicates). Bar = 10 μm.


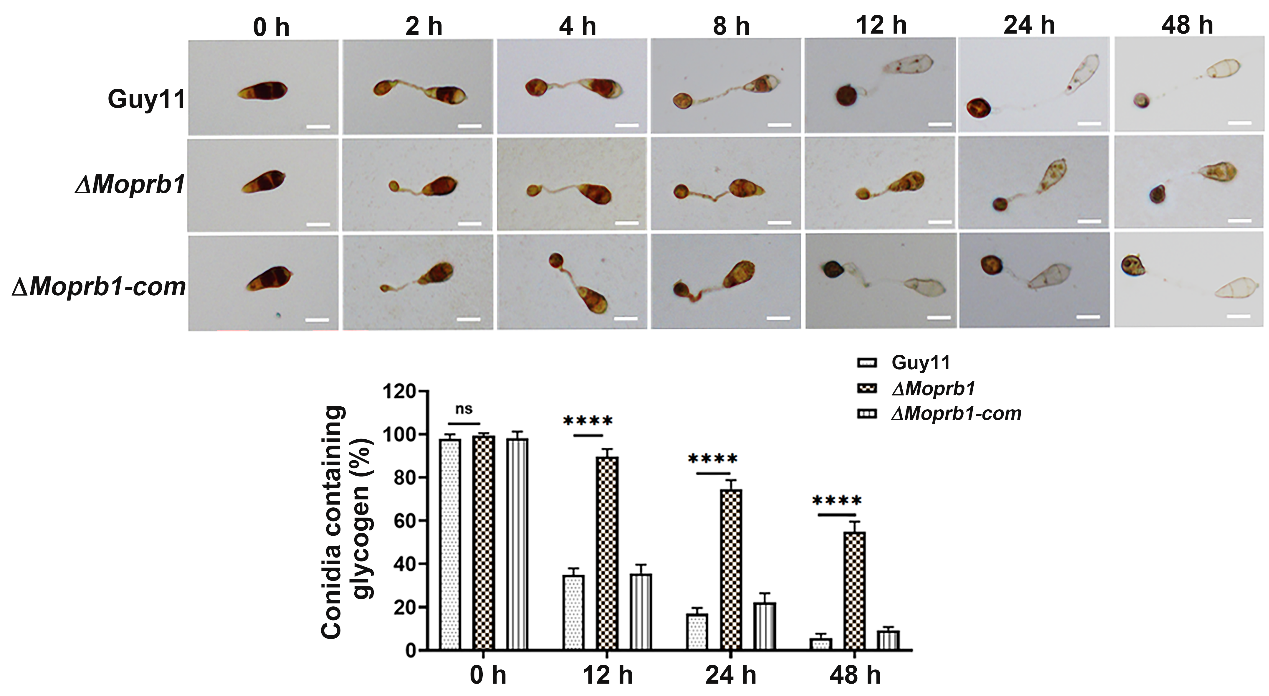


**Fig. S7 Deletion of *MoPRB1* impairs turnover of glycogen during conidial germination and appressorium morphogenesis.** Conidia from the wild type (Guy11), Δ*Moprb1* and the complemented strain (Δ*Moprb1-com*) were germinated on hydrophobic Gelbond membranes. Drop of water was replaced by iodine solution (60 mg/mL of KI and 10 mg/mL of I_2_ in distilled water) at 0 h, 2 h, 4 h, 8 h, 12 h, 24 h and 48 h to stain the spores for 2 minutes and microscopically visualize glycogen as yellowish-brown deposits. Bar = 10 µm. The bar chart shows the average number of conidia containing glycogen at each time point. At least 100 conidia were counted per biological replication and the experiment was performed in triplicate. Means and standard deviations were calculated from three independent biological replicates. ^ns^, *P* > 0.05; ****, *P* < 0.0001.

**
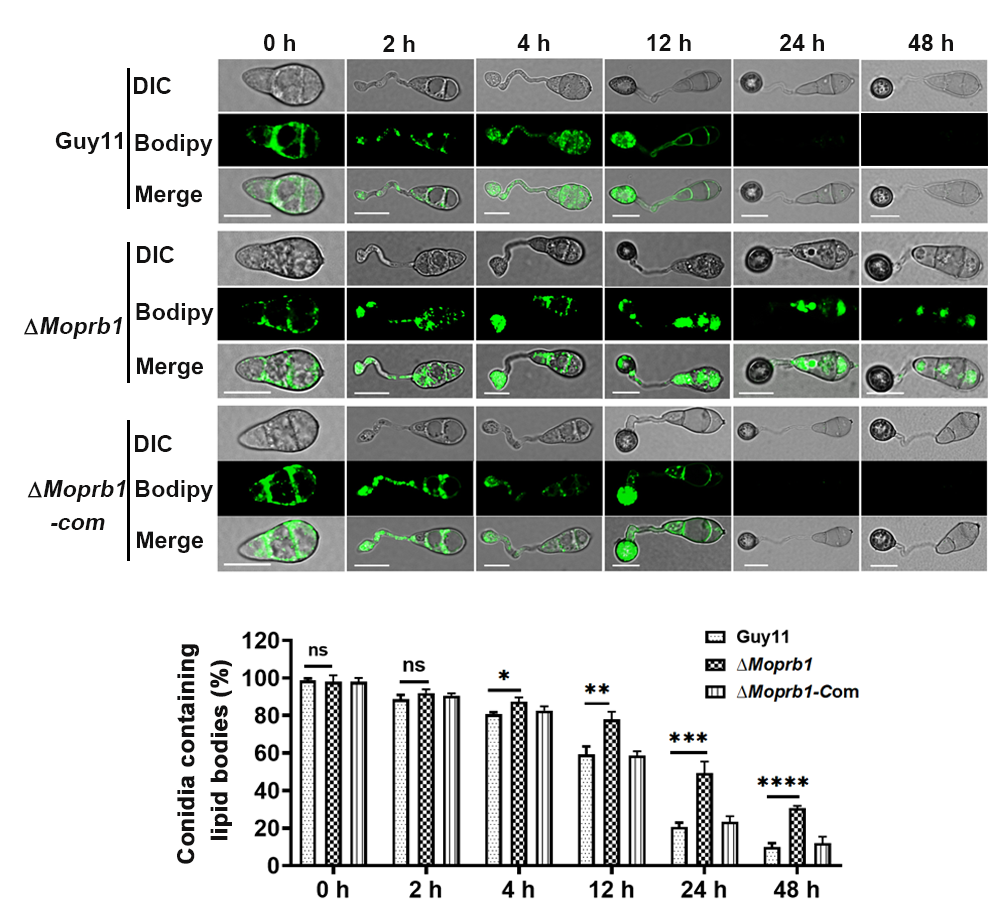
**

**Fig. S8 MoPrb1 is involved in lipid body translocation and degradation during appressorium morphogenesis.** Conidia of Guy11, Δ*Moprb1* and Δ*Moprb1-com* were incubated in water droplets on the hydrophobic surface of Gelbond and allowed to form appressoria for up to 48 h. Samples were removed at 0, 2, 4, 12, 24 and 48 h and stained with Bodipy (D3922, Invitrogen) to visualize lipid bodies by confocal microscopy. Bar = 10 µm. The bar chart shows the average number of conidia containing lipid bodies at each time point. At least 100 conidia were counted per biological replication and the experiment was performed in triplicate. Means and standard deviations were calculated from three independent biological replicates. ^ns^, *P* > 0.05; ^*^, *P* < 0.05; **, *P* < 0.01; ***, *P* < 0.001; ****, *P* < 0.0001.

*
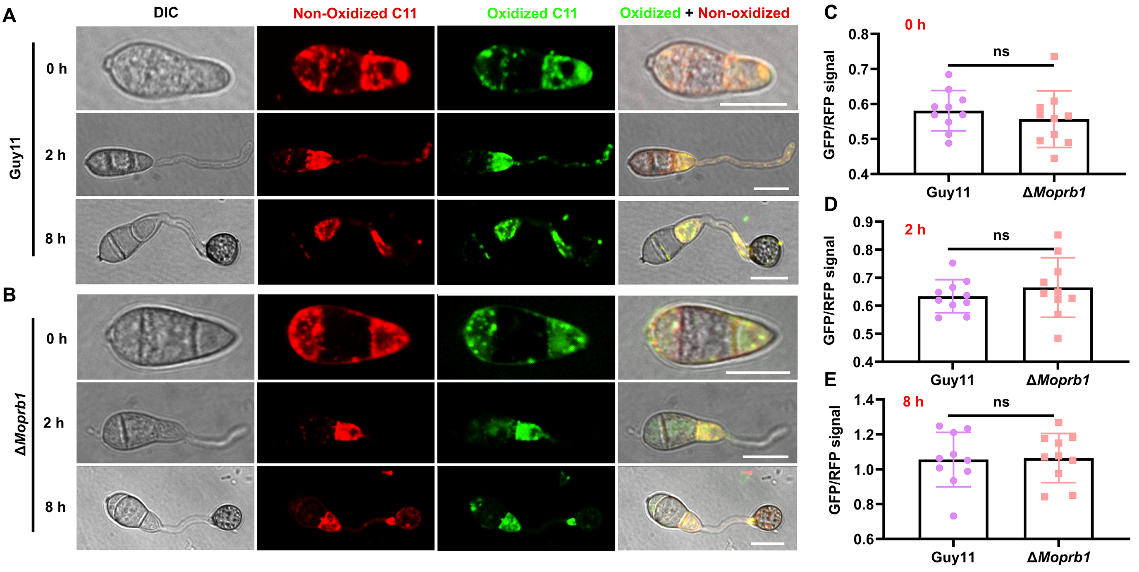
*

**Fig. S9 Ferroptosis is not disrupted in the Δ*Moprb1* mutant at the various developmental stages of *M. oryzae*.** (A, B) Conidia were incubated on hydrophobic coverslips for 0, 2 and 8 hours, then stained with 10 μM C11-BODIPY^581/591^ for 30 minutes to visualize and quantify oxidized and unoxidized lipid peroxides. Bar = 10 μm. (C-E) Lipid peroxidation levels were determined through quantitative analysis by calculating the pixel intensity ratio of GFP (oxidized form) to RFP (unoxidized form) signals, with at least 10 replicates analyzed (n ≥ 10). ^ns^, *P* > 0.05. Data are presented as mean ± SD.


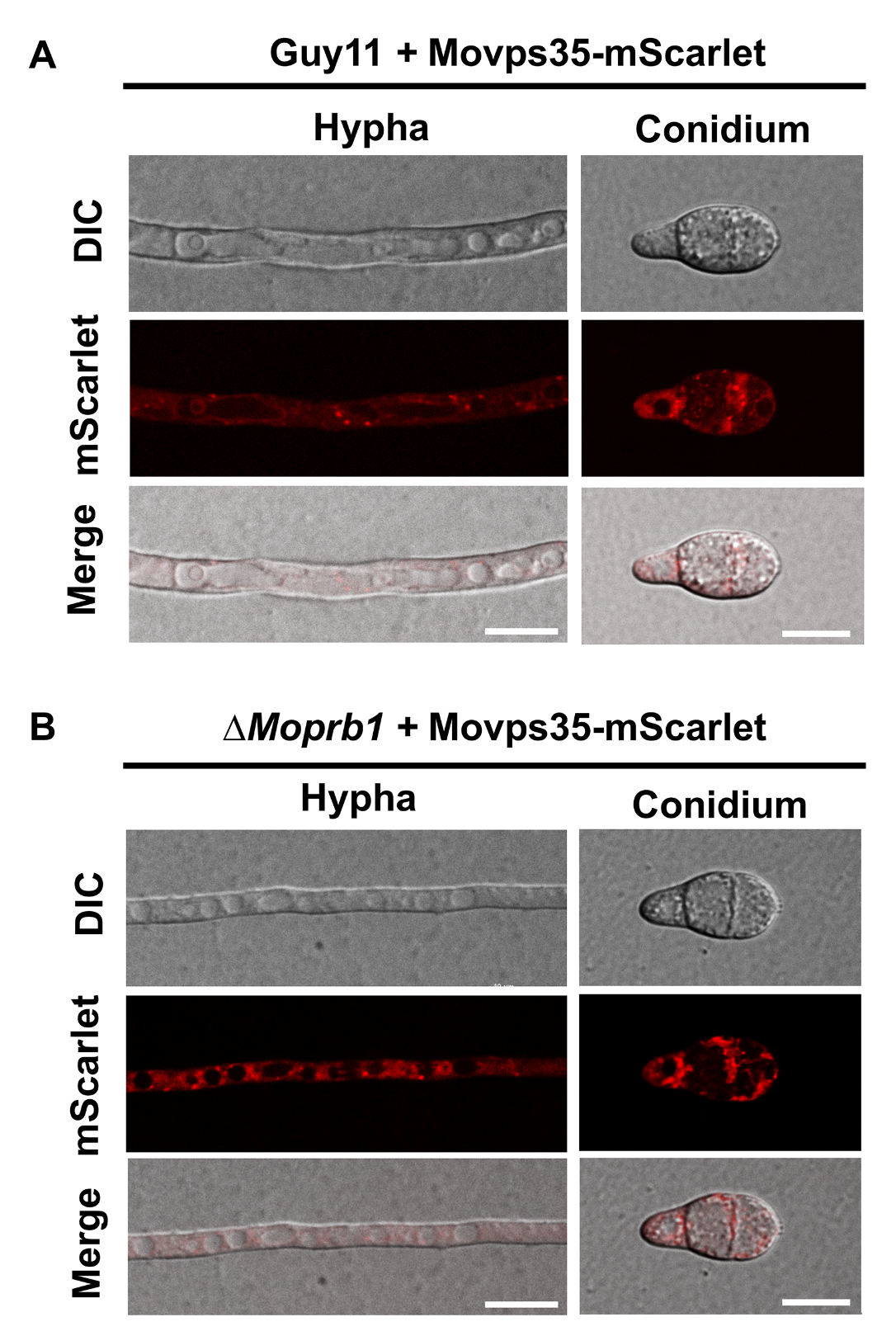


**Fig. S10 Deletion of *MoPrb1* does not affect the subcellular localization of MoVps35.** (A) Subcellular localization of MoVps35-mScarlet in Guy11. (B) Subcellular localization of MoVps35-mScarlet in the *∆Moprb1* mutant. Bar = 10 μm.

**
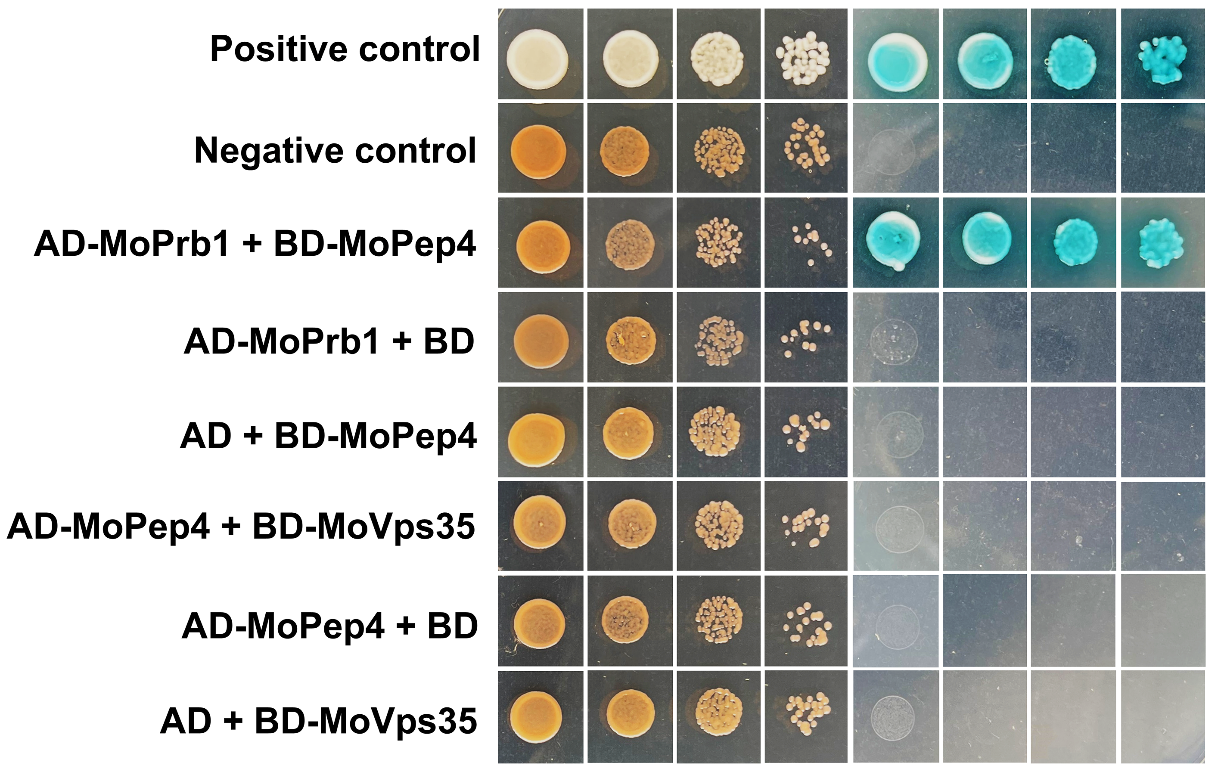
**

**Fig. S11** **The vacuolar aspartyl protease MoPep4 directly interacts with MoPrb1 but not with MoVps35.** Yeast two-hybrid assays were performed to verify the interactions of MoPep4 with MoPrb1 and MoVps35. Transformants were grown on SD/-Leu-Trp and SD/-Leu-Trp-His-Ade media supplemented with X-α-gal, respectively. pGADT7-T + pGBKT7-53 and pGADT7-T + pGBKT7-Lam were used as positive and negative controls, respectively.


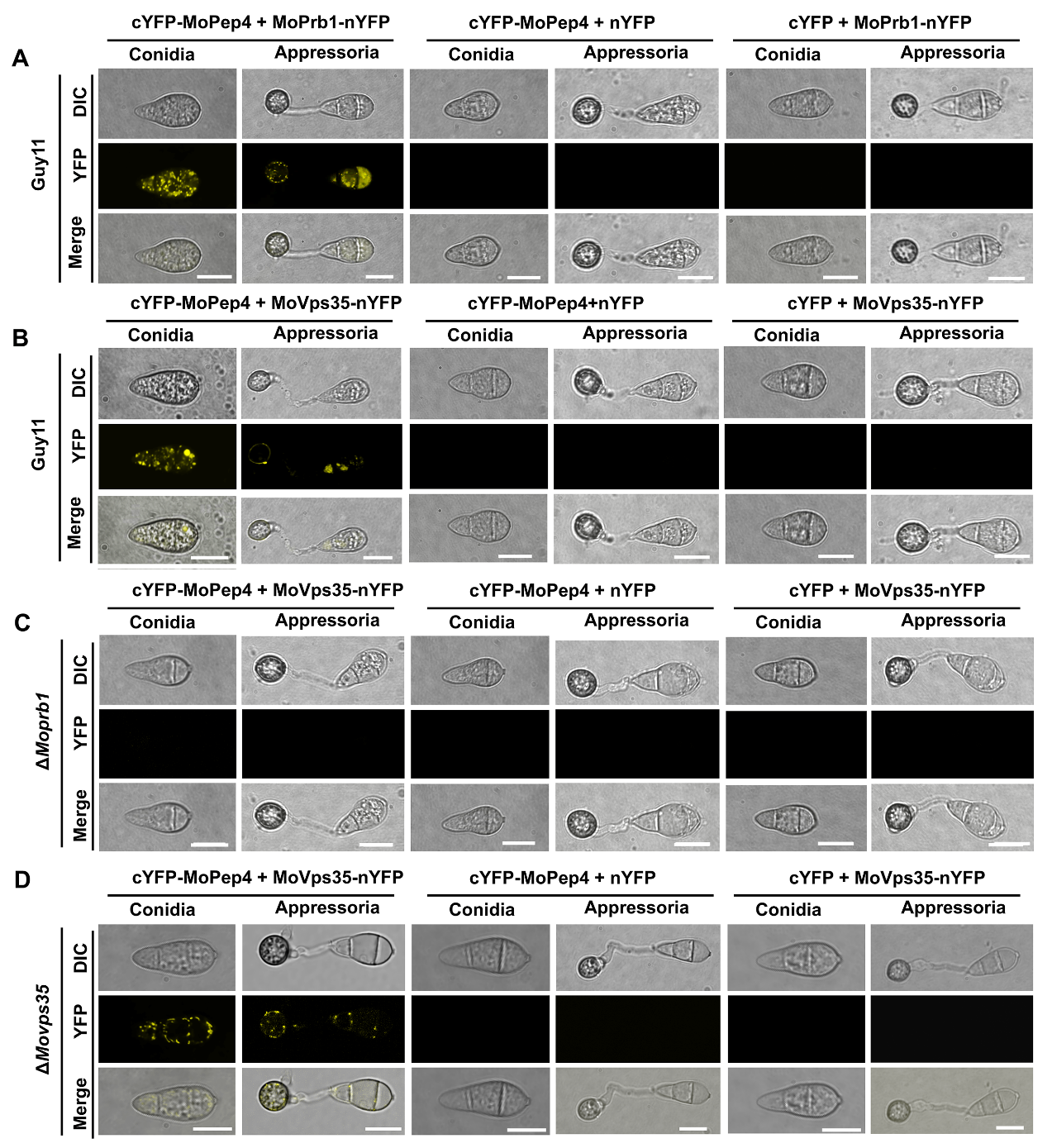


**Fig. S12 The vacuolar aspartyl protease MoPep4 directly interacts with MoPrb1 and indirectly with MoVps35 via MoPrb1.** (A, B) Bimolecular fluorescence complementation (BiFC) assay was used to visualize the interactions between MoVps35, MoPrb1 and MoPep4 in vivo. In the Guy11, reconstituted YFP signals were observed in strains expressing the cYFP-MoPep4/MoPrb1-nYFP constructs as well as in strains expressing the cYFP-MoPep4/MoVps35-nYFP constructs. However, (C) in the Δ*Moprb1* mutant, no reconstituted YFP signal was detected in strains expressing the cYFP-MoPep4/MoVps35-nYFP constructs. (D) In the Δ*Movps35* mutant, reconstituted YFP signals were observed in strains expressing the cYFP-MoPep4/MoPrb1-nYFP constructs, while YFP signals were not detected in the negative control strains. Bar = 10 μm.


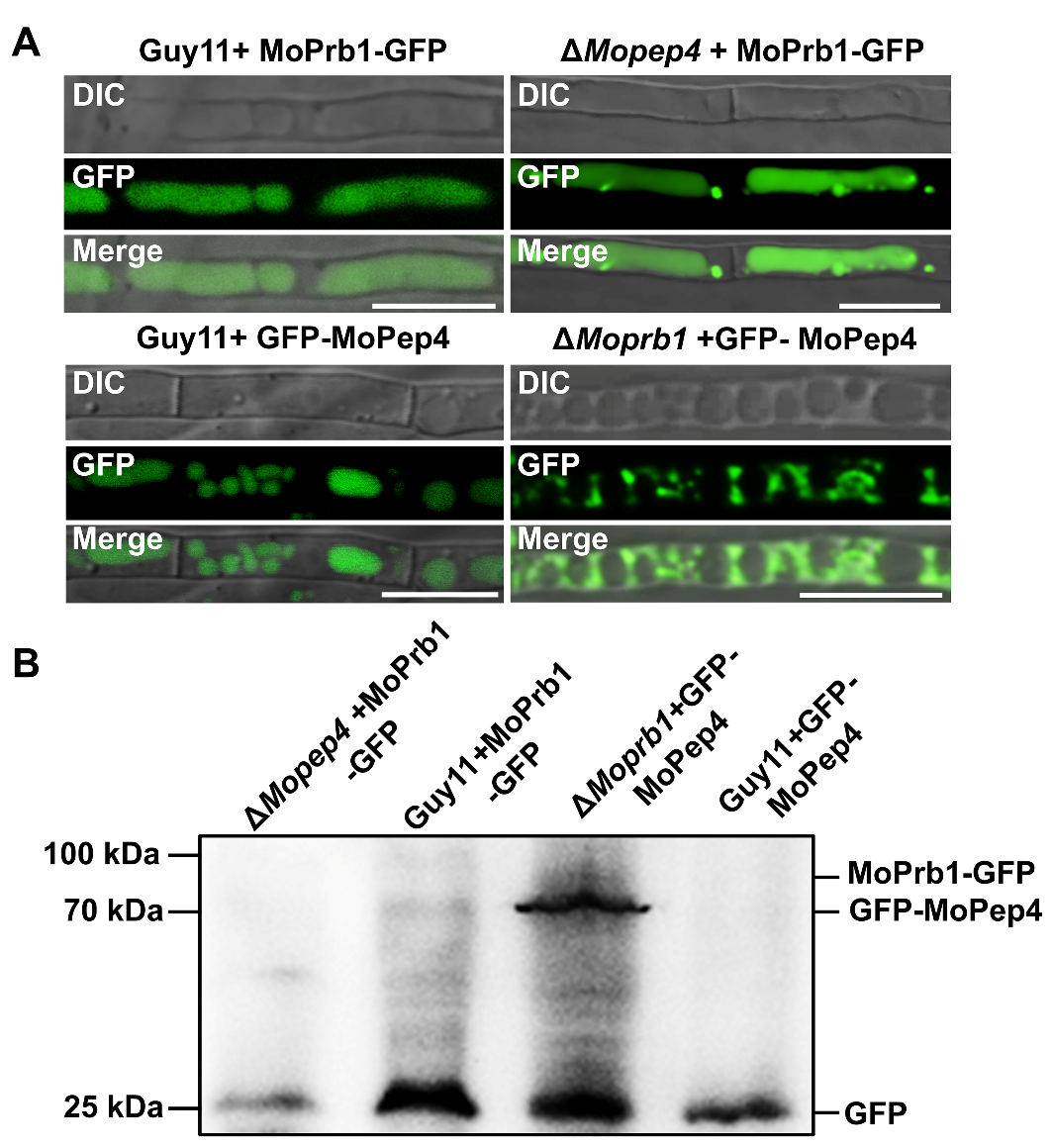


**Fig. S13** (A) Subcellular localization of MoPrb1-GFP in the Δ*Mopep4* mutant and GFP- MoPep4 in the Δ*Moprb1* mutant. (B) Western blot analysis showed that MoPrb1 is required for MoPep4 function through its regulation and modification.


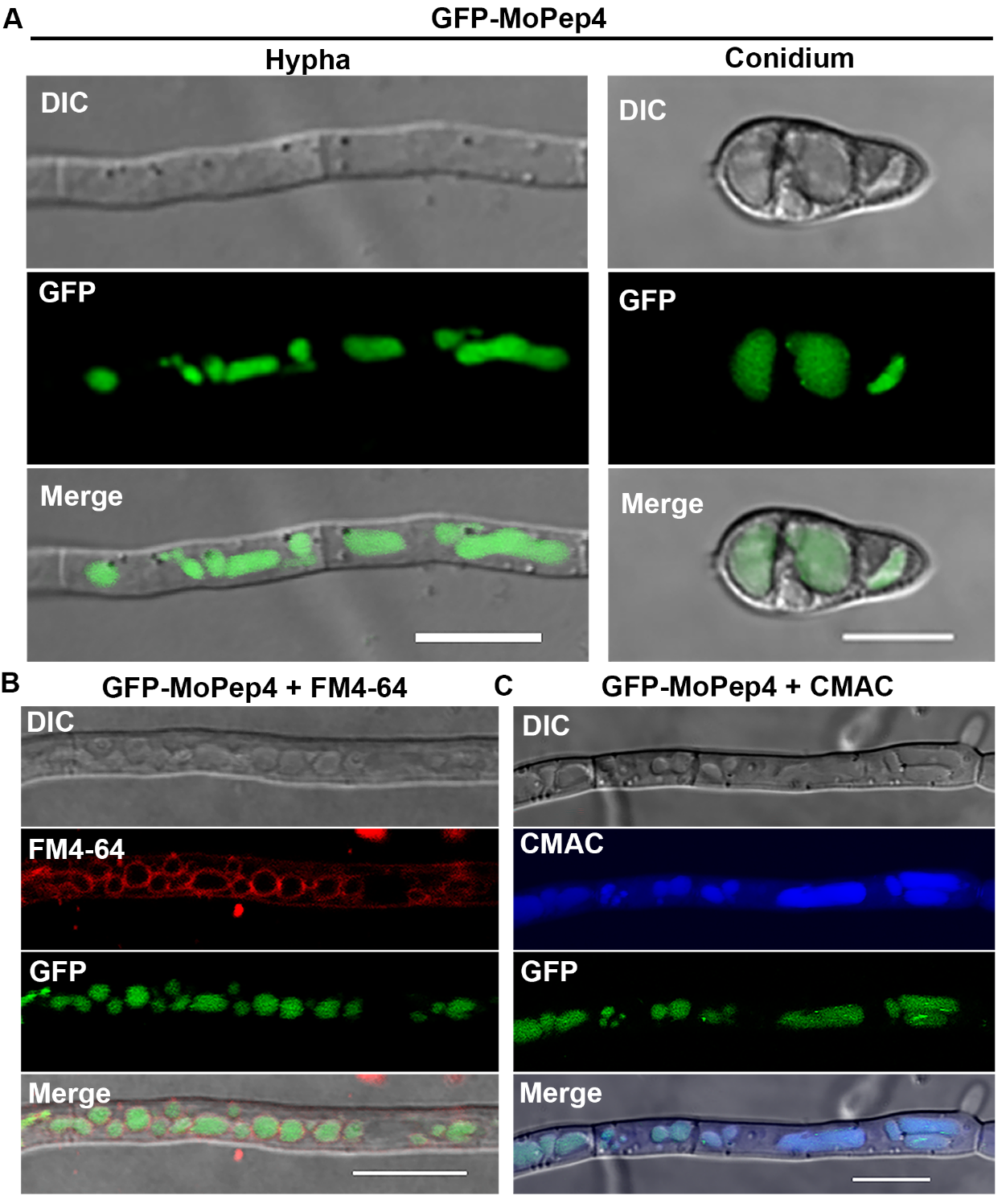


**Fig. S14 Subcellular localization of GFP-MoPep4.** (A) Subcellular localization of GFP-MoPep4 during morphogenesis process. GFP-MoPep4 was observed to be mainly localized in the vacuole. Bar = 10 μm. (B) Mycelia expressing GFP-MoPep4 were stained with FM4-64 (a vacuolar membrane dye) and examined using a confocal microscope. Bar = 10 μm. (C) Mycelia expressing GFP-MoPep4 were stained with CMAC (a vacuolar lumen dye) and examined using a laser confocal microscope. Bar = 10 μm.


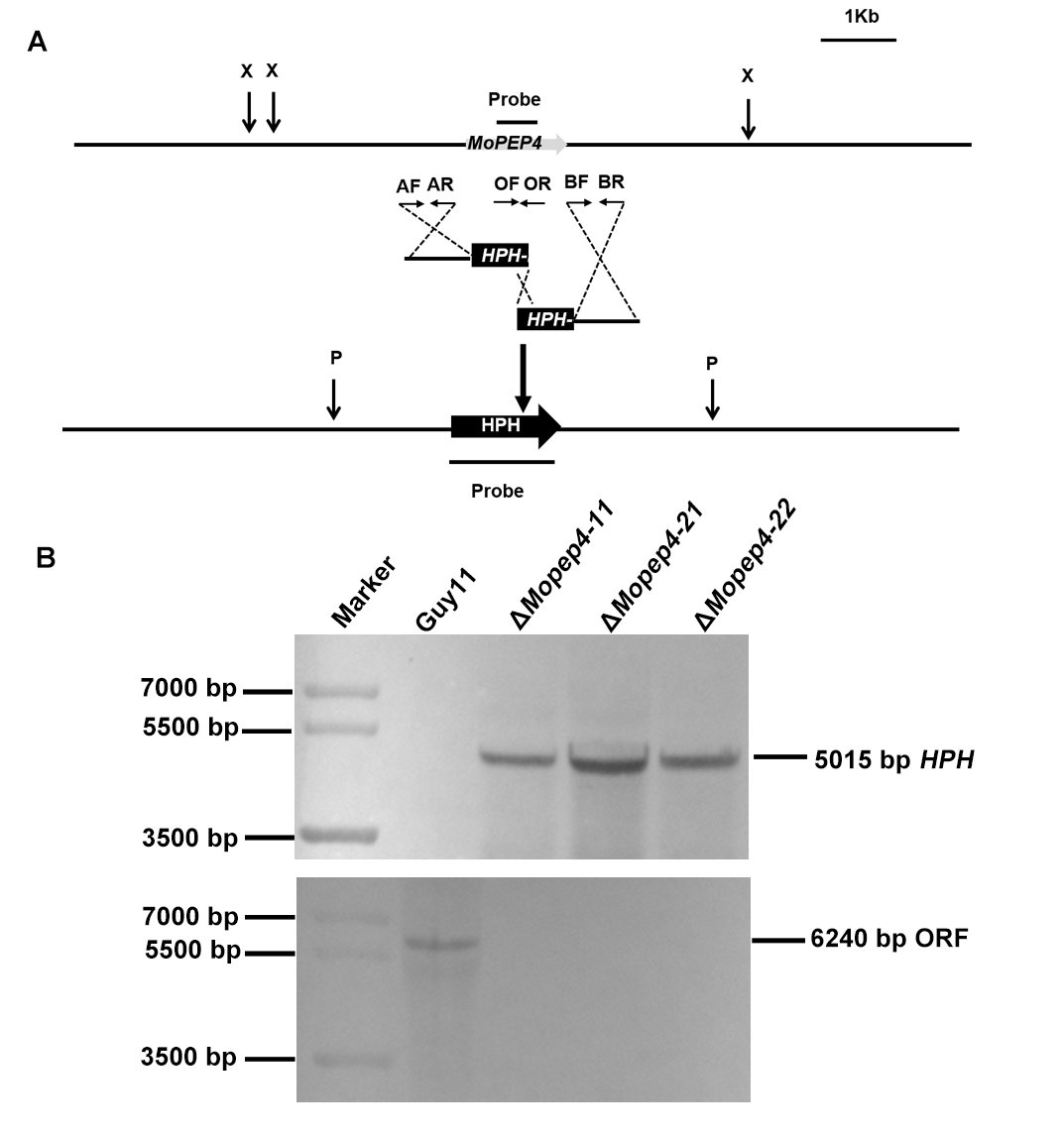


**Fig. S15 *MoPEP4* gene deletion strategy and confirmation.** (A) Gene deletion strategy for *MoPEP4.* (B) Southern blot confirmation for *MoPEP4* gene deletion. *Pst*I was used for genomic DNA digestion, and the hygromycin B phosphotransferase (*HPH*) gene was amplified (using the primer pair HPH-F / HPH-R) and used as probe 1. The 5.0 kb target band was detected in the *∆Mopep4* mutants, while no band was detected in Guy11. *Xho*I was also used for genomic DNA digestion and the partial open reading frame (ORF) of the *MoPEP4* gene was amplified (using the primer pair OF/OR) and used as probe 2. A 6.2 kb target band was detected in Guy11, while no band was detected in the *∆Mopep4* mutants as predicted.


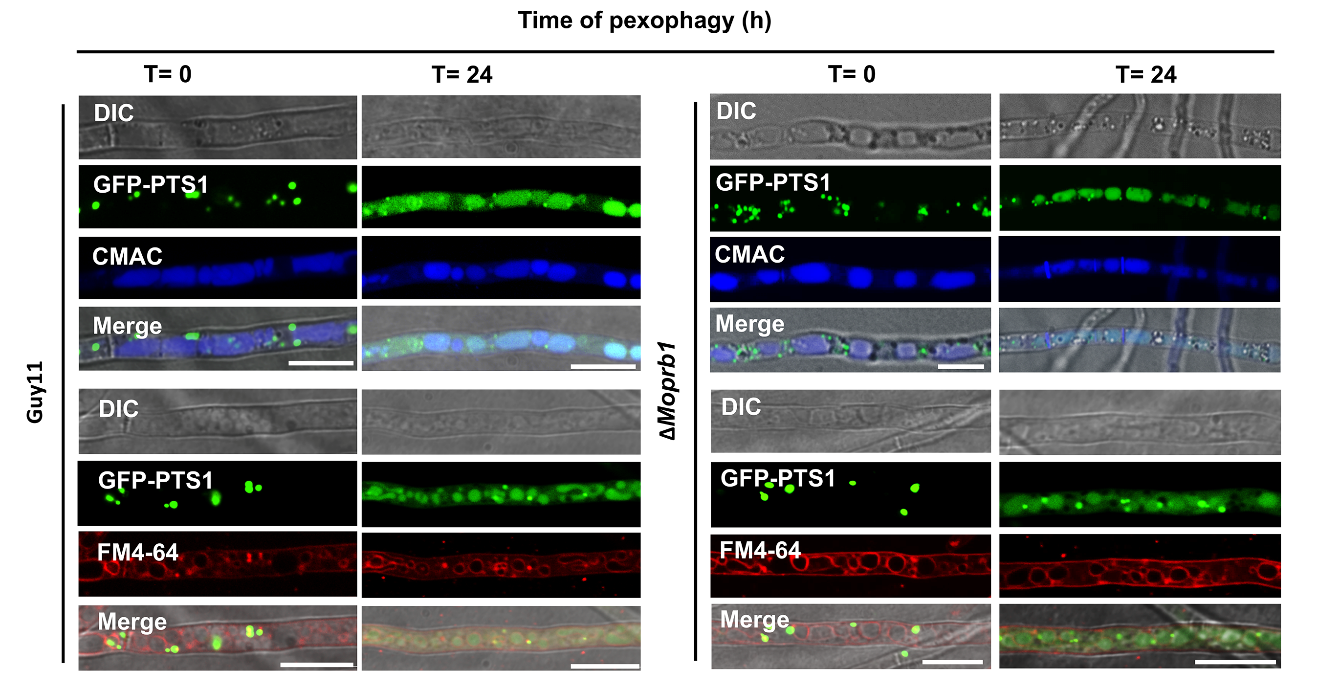


**Fig. S16 MoPrb1 is dispensable for pexophagy.** Peroxisomes were labeled with the matrix protein GFP-PTS1, which is aare peroxisome marker. Prior to the induction of pexophagy (T = 0 h), the peroxisomes were visible as green cytosolic dots and the vacuolar lumen did not display any signals. After induction of pexophagy (T = 24 h), some peroxisomal signals remained in the cytosol, while the vacuoles of Guy11 and Δ*Moprb1* were was filled with diffuse green fluorescence signals, indicating peroxisomal breakdown. The vacuoles and membrane were stained with CMAC and FM4-64. Bar = 10 μm.

**Table S1 Interacting partners of MoVps35 identified by IP-MS（see excel file）**

**Table S2 The fungal strains used in this study**

| **Strain** | | **Genotype description** | **Reference** | |
| --- | --- | --- | --- | --- |
| Guy11 | | Wild-type | | ^1^ |
| Δ*Moprb1* | | *MoPRB1* deletion mutant of Guy11 | | This study |
| Δ*Moprb1-*com | | Δ*Moprb1* expressing MoPrb1-GFP construct | | This study |
| Δ*Movps35* | | *MoVPS35* deletion mutant of Guy11 | | ^1^ |
| MoPrb1-nYFP+MoVps35-cYFP | | Guy11 transformant expressing MoPrb1-nYFP and MoVps35-cYFP | | This study |
| MoPrb1-nYFP+pCX62-cYFP | | Guy11 transformant expressing MoPrb1-nYFP and pCX62-cYFP | | This study |
| MoVps35-cYFP+pKNT-nYFP | | Guy11 transformant expressing MoVps35-cYFP and pKNT-nYFP | | This study |
| MoPep4-cYFP+MoVps35-nYFP | | Guy11 transformant expressing MoPep4-cYFP and MoVps35-nYFP | | This study |
| MoPep4-cYFP+ pKNT-nYFP | | Guy11 transformant expressing MoPep-cYFP and pKNT-nYFP | | This study |
| pCX62-cYFP+ MoVps35-nYFP | | Guy11 transformant expressing cYFP and MoVps35-nYFP | | This study |
| MoPep4-cYFP+MoPrb1-nYFP | | Guy11 transformant expressing MoPep4-cYFP and MoPrb1-nYFP | | This study |
| Δ*Moprb1/* MoPep4-cYFP+MoVps35-nYFP | | Δ*Moprb1* transformant expressing MoPep4-cYFP and MoVps35-nYFP | | This study |
| Δ*Moprb1/* pCX62-cYFP+MoVps35-nYFP | | Δ*Moprb1* transformant expressing pCX62-cYFP and MoVps35-nYFP | | This study |
| Δ*Moprb1/* MoPep4-cYFP+ pKNT-nYFP | | Δ*Moprb1* transformant expressing MoPep4-cYFP and pKNT-nYFP | | This study |
| Δ*Movps35/* MoPep4-cYFP+ nYFP | | Δ*Movps35* transformant expressing MoPep4-cYFP and pKNT-nYFP | | This study |
| Δ*Movps35/* pCX62-cYFP+MoPrb1-nYFP | | Δ*Movps35* transformant expressing pCX62-cYFP and MoPrb1-nYFP | | This study |
| Δ*Movps35/* MoPep4-cYFP+MoPrb1-nYFP | | Δ*Movps35* transformant expressing MoPep4-cYFP and MoPrb1-nYFP | | This study |
| Guy11+GFP-MoAtg8 | | Guy11 transformant expressing GFP-MoAtg8 | | This study |
| Δ*Moprb1*+GFP-MoAtg8 | | Δ*Moprb1* transformant expressing GFP-MoAtg8 | | This study |
| Guy11+MoPrb1-GFP | | Guy11 transformant expressing MoPrb1-GFP | | This study |
| Δ*Movps35*+MoPrb1-GFP | | Δ*Movps35* transformant expressing MoPrb1-GFP | | This study |
| Δ*Movps29*+MoPrb1-GFP | | Δ*Movps29* transformant expressing MoPrb1-GFP | | This study |
| Δ*Movps26*+MoPrb1-GFP | | Δ*Movps26* transformant expressing MoPrb1-GFP | | This study |
| Δ*Movps17*+MoPrb1-GFP | | Δ*Movps17* transformant expressing MoPrb1-GFP | | This study |
| Movps35-GFP+MoPrb1-mCherry | | Guy11 transformant expressing MoVps35-GFP and MoPrb1-mCherry | | This study |
| Δ*Mopep4* | | *MoPEP4* deletion mutant of Guy11 | | This study |
| Δ*Mopep4*+GFP-MoAtg8 | Δ*Mopep4* transformant expressing GFP-MoAtg8 | | | This study |
| Guy11+GFP-MoPep4 | Guy11 transformant expressing GFP-MoPep4 | | | This study |
| Δ*Movps35*+GFP-MoPep4 | Δ*Movps35 t*ransformant expressing GFP-MoPep4 | | | This study |
| Δ*Movps29*+GFP-MoPep4 | Δ*Movps29* transformant expressing GFP-MoPep4 | | | This study |
| Δ*Movps26*+GFP-MoPep4 | Δ*Movps26* transformant expressing GFP-MoPep4 | | | This study |
| Δ*Movps17*+GFP-MoPep4 | Δ*Movps17* transformant expressing GFP-MoPep4 | | | This study |
| *ΔMoprb1ΔMopep4* | Double gene deletion of *MoPRB1* and *MoPEP4* | | | This study |
| GFP-PTS1+Guy11 | Guy11 expressing GFP-PTS1 construct | | | This study |
| GFP-PTS1+Δ*Moprb1* | *ΔMoprb1* transformant expressing GFP-PTS1 | | | This study |
| GFP-PTS1+Δ*Mopep4* | *ΔMopep4* transformant expressing GFP-PTS1 | | | This study |
| Guy11+MoVps35-mScarlet | Guy11 transformant expressing MoVps35-mScarlet | | | This study |
| Δ*Moprb1*+MoVps35-mScarlet | *ΔMoprb1* transformant expressing MoVps35-mScarlet | | | This study |
| *ΔMoprb1ΔMopep4/MoPRB1* | *ΔMoprb1ΔMopep4* transformant expressing GFP-MoPep4 | | | This study |
| Guy11+H4-GFP | Guy11 transformant expressing H4-GFP | | | This study |
| Δ*Moprb1*+H4-GFP | Δ*Moprb1* transformant expressing H4-GFP | | | This study |
| Δ*Moatg8*+H4-GFP | Δ*Moatg8* transformant expressing H4-GFP | | | This study |
| Δ*Moprb1*+ GFP-MoPep4 | Δ*Moprb1* transformant expressing GFP-MoPep4 | | | This study |
| Δ*Mopep4*+ MoPrb1-GFP | Δ*Mopep4* transformant expressing MoPrb1-GFP | | | This study |
| Δ*Moprb1/*Δ*Moprb1*^D192A H224A S390A^ | Δ*Moprb1* transformant expressing MoPrb1^D192A H224A S390A^ - mCherry and GFP-MoAtg8 | | | This study |
| Δ*Moprb1*Δ*Mopep4/*Δ*MoPRB1* | Δ*Moprb1*Δ*Mopep4*Δ*Moprb1* transformant expressing GFP-MoPep4 | | | This study |
| Guy11+MoPex14-GFP | Guy11 transformant expressing MoPex14-GFP | | | This study |
| Δ*Mopep4*+MoPex14-GFP | Δ*Mopep4* transformant expressing MoPex14-GFP | | | This study |

**Table S3 Plasmids used in this study**

| **Plasmids** | **Description** |
| --- | --- |
| MoPrb1-nYFP | *MoPRB1* (MGG_03670) promoter and entire coding sequence with a C-terminal translational fusion of the cYFP reporter gene. A 2.5-kb *MoPRB1* gene fragment cloned into KpnI-HindIII restriction sites of pKNT-NYFP (GeneticinR, AmpicillinR) |
| MoVps35-cYFP | *MoVPS35* (MGG_05089) promoter and entire coding sequence with a C-terminal translational fusion of the nYFP reporter gene. A 3.5-kb *MoVPS35* gene fragment cloned into KpnI-HindIII restriction sites of pCX62-CYFP (HygromycinR, AmpicillinR) |
| GFP-MoAtg8 | A *MoATG8* cDNA fragment with the terminator codon was amplified and inserted into the *SpeI* site of p1300RPGFP to generate pGFP-MoATG8. Published in Liu et al., 2015^2^ |
| AD-MoVps35 | *MoVPS35* (MGG_05089) cDNA fragment was cloned into yeast expression vector pGADT7 |
| BD-MoVps35 | *MoVPS35* (MGG_05089) cDNA fragment was cloned into yeast expression vector pGBKT7 |
| AD-MoPrb1 | *MoPRB1* (MGG_03670) cDNA fragment was cloned into yeast expression vector pGADT7 |
| BD-MoPrb1 | *MoPRB1* (MGG_03670) cDNA fragment was cloned into yeast expression vector pGBKT7 |
| AD-MoPep4 | *MoPEP4* (MGG_00922) cDNA fragment was cloned into yeast expression vector pGADT7 |
| BD-MoPep4 | *MoPEP4* (MGG_00922) cDNA fragment was cloned into yeast expression vector pGBKT7 |
| MoVps35-GFP | *MoVPS35* (MGG_05089) promoter and entire coding sequence with a C-terminal translational fusion of the GFP reporter gene. Published in Zheng et al., 2015 |
| MoPrb1-mCherry | *MoPRB1* (MGG_03670) promoter and entire coding sequence with a C-terminal translational fusion of the mCherry reporter gene. The *MoPRB1* gene fragment cloned into KpnI-HindIII restriction sites of pKNT-mCherry (GeneticinR, AmpicillinR) |
| MoPrb1-GFP | *MoPRB1* (MGG_03670) promoter and entire coding sequence with a C-terminal translational fusion of the GFP reporter gene |
| GFP-MoPep4 | *MoPEP4* (MGG_00922) promoter and entire coding sequence with a N-terminal translational fusion of the GFP reporter gene |
| GFP-PTS1 | RP27 promoter and the GFP ORF with PTS1 signal (serine-lysine-leucine, SKL) |
| MoVps35-mScarlet | *MoVPS35* (MGG_05089) promoter and entire coding sequence with a C-terminal translational fusion of the mScarlet gene |
| H4-GFP | *Magnaporthe oryzae 70-15* histone H4 (MGG_06293) promoter and entire coding sequence with a C-terminal translational fusion of the GFP gene |
| MoPrb1^D192A H224A S390A^ - mCherry | *The MoPRB1 (MGG_03670)* promoter and the mutant MoPRB1 coding sequence, in which the 192D, 224H, and 390S sites were mutated to 192A, 224A, and 390A, respectively, were fused at the C-terminus with the mCherry reporter gene in a translational fusion. The mutated MoPRB1 gene fragment cloned into KpnI-HindIII restriction sites of pKNT-mCherry (GeneticinR, AmpicillinR) |
| MoPep4-cYFP | *MoPEP4* (MGG_00922) promoter and entire coding sequence with a C-terminal translational fusion of the nYFP reporter gene. A 5.0 -kb *MoPEP4* gene fragment cloned into KpnI-HindIII restriction sites of pCX62-CYFP (HygromycinR, AmpicillinR) |
| MoVps35-nYFP | *MoVPS35* (MGG_05089) promoter and entire coding sequence with a C-terminal translational fusion of the nYFP reporter gene. A 3.5-kb *MoVPS35* gene fragment cloned into KpnI-HindIII restriction sites of pKNT-NYFP (GeneticinR, AmpicillinR) |
| MoPex14-GFP | RP27 promoter and *MoPEX14* (MGG_01028) entire coding sequence with a C-terminal translational fusion of the GFP reporter gene. The gene fragment containing the RP27 promoter and *MoPEX14* was cloned into XhoI- HindIII restriction sites of pKNT -RP27-GFP (GeneticinR, AmpicillinR) |

**Table S4 PCR** **primers used in this study**

| **Primers** | **Sequence（5'-3'）** | **Application** |
| --- | --- | --- |
| MoPRB1-ADF | GCCATGGAGGCCAGTGAATTCATGAAGAGCGTCATCCTCCTT | Construct AD-MoPrb1 vector |
| MoPRB1-ADR | CAGCTCGAGCTCGATGGATCCTTACACGCGAGCCTCACC |  |
| MoVPS35-BDR | CATGCCATGGAGATGGCGTCGGTCCCAGCTC | Construct BD-MoVps35 vector |
| MoVPS35-BDR | CATGCCATGGTCACTTGGGATCCAACACAATTCC |  |
| MoPRB1_BiFC-F | AGGGAACAAAAGCTGGGTACCAAGTGGCGATCTTTACGAAT | Construct nYFP-MoPrb1 |
| MoPRB1_BiFC-R | CGTGGCGATGGAGCGAAGCTTCACGCGAGCCTCACCGAAAAA |  |
| MoVPS35_BiFC-F | GGGAACAAAAGCTGGGTACCATGGCGTCGGTCCCAGCTC | Construct cYFP-MoVps35 |
| MoVPS35_BiFC-R | TTGCAGGCCGGGCGAAGCTTTCACTTGGGATCCAACACAATTCC |  |
| MoPRB1-AF | TACCCAACTCCATCCCAATT | *MoPRB1* deletion and verification |
| MoPRB1-AR | TTGACCTCCACTAGCTCCAGCCAAGCCTGCCTGCGACCAAACAA |  |
| MoPRB1-OF | TTCATTGATTGTCATTGGGTG |  |
| MoPRB11-OR | GCGGCGGGAGAATAGTT |  |
| MoPRB1-BF | GAATAGAGTAGATGCCGACCGCGGGTTATGTCTACCGGGTTTGGAG |  |
| MoPRB1-BR | CTGTTGAGTACGACGAGGGA |  |
| MoPRB1-UA | ATCACCTGAAGATGGGAGACA |  |
| H853 | GACAGACGTCGCGGTGAGTT |  |
| MoPRB1-CF | AGGGAACAAAAGCTGGGTACCGGGATAGGATAGGCACAA | Complementation and GFP tagging |
| MoPRB1-CR | GCCCTTGCTCACCATAAGCTTCACGCGAGCCTCACCGAAA |  |
| GFP-F | CACAAGTTCAGCGTGTCCG | GFP verification |
| GFP-R | AGTTCACCTTGATGCCGTTC |  |
| RP27-GFP-PTS1-F | AGGGAACAAAAGCTGGGTACCATAAATGTAGGTATTACCTGTAC | Construct pKNT-GFP-PTS1 vector |
| RP27-GFP-PTS1-R | TTAAGTGGATCCCCCGGGTTACAGCTTCGACTTGTACAGCTCGTCCATG |  |
| MoPRB1-MF | GTCGACGGTATCGATAAGCTTGGGATAGGATAGGCACAAG | Construct MoPrb1-mCherry |
| MoPRB1-MR | TCCTCGCCCTTGCTCACCATCCCGGGCACGCGAGCCTCACCGAA |  |
| MoPEP4-ADF | GCCATGGAGGCCAGTGAATTCATGAAGGGAGCTATGATGAC | Construct AD- MoPep4 |
| MoPEP4-ADR | CAGCTCGAGCTCGATGGATCCTTATTTGGCCTTGGCCAGGC |  |
| MoPEP4-BDF | ATGGCCATGGAGGCCGAATTCATGAAGGGAGCTATGATGAC | Construct BD-MoPep4 |
| MoPEP4-BDR | CCGCTGCAGGTCGACGGATCCTTATTTGGCCTTGGCCAGGC |  |
| MoPEP4-AF | AGGTGGTAGGTGGTAGAGCG | *MoPEP4* deletion and verification |
| MoPEP4-AR | TTGACCTCCACTAGCTCCAGCCAAGCCTCGGGATTGCGAACAGAA |  |
| MoPEP4-OF | CCTGTTGAGCCCTACTATCC |  |
| MoPEP4-OR | TTGTTGACACCACCAAAGAC |  |
| MoPEP4-BF | GAATAGAGTAGATGCCGACCGCGGGTTTGCGGATGGTTGTCAATT |  |
| MoPEP4-BR | CGAGGGATGCTAGGTAGG |  |
| MoPEP4-UA | TTGACCTTGTTACATTGAGT |  |
| H853 | GACAGACGTCGCGGTGAGTT |  |
| HYG/F | GGCTTGGCTGGAGCTAGTGGAGGTCAA | N-terminal portion of the *HPH* cassette |
| HY/R | GTATTGACCGATTCCTTGCGGTCCGAA |  |
| YG/F | GATGTAGGAGGGCGTGGATATGTCCT | C-terminal portion of the *HPH* cassette |
| HYG/R | AACCCGCGGTCGGCATCTACTCTATTC |  |
| GFP-MoPEP4_F | ACCCAATCTTCAAACTCGAG ATGGTGAGCAAGGGCGAG | Complementation and GFP tagging |
| GFP-MoPEP4_R | CAGTAACGTTAAGTGGATCCTTATTTGGCCTTGGCCAGG |  |
| GFP-MoPEP4_SoeF | TACAAGCCCGGGAAGCTTATGAAGGGAGCTATGATGAC |  |
| GFP-MoPEP4_SoeR | CTTCATAAGCTTCCCGGGCTTGTACAGCTCGTCCATG |  |
| MoVPS35-mScarlet-F | GGGAACAAAAGCTGGGTACCATGGCGTCGGTCCCAGCTC | Construct MoVps35-mScarlet vector |
| MoVPS35-mScarlet-R | GCCCTTGCTCACCATAAGCTTTCACTTGGGATCCAACACAATTCC |  |
| H4pro-KpnIF | GGAACAAAAGCTGGGTACCACAGAGTCTCTGCTCGTGCGA | Construct H4-GFP vector |
| H4pro-XhoIR | CGATACCGTCGACCTCGAGCTTGATGGATTTGAATGAGAA |  |
| H4cds-XhoIF | CAAATCCATCAAGCTCGAGATGACTGGACGCGGAAAGGGA |  |
| H4cds-gfpXhoIR | GATACCGTCGACCTCGAGACCACCGAAACCGTAGAGGGT |  |
| MoPEP4-A1F | TACGCCAGCCAGGTGATA | Deletion of *MoPEP4* on the background of Δ*Moprb1* |
| MoPEP4-A1R | TGGAAATTGTAAGCGTTAATCTAGAGGAGAATGCAAGCCCAAA |  |
| MoPEP4-B1F | TCGCCTTCTTGACGAGTTCTTCTGATATGGGCAGTAGGTAGTGGTAC |  |
| MoPEP4-B1R | ATGGCAACGAATGTCAGCA |  |
| MoPEP4-OF1 | CATCGTCTTGCGGGTCTA |  |
| MoPEP4-OR1 | AGGGCAATGAGCGAAGTG |  |
| MoPEP4-UF1 | TAGCCGACAATGCGTAAGTA |  |
| MoPEP4-UR1 | GCAGGAGCAAGGTGAGATG |  |
| MoPRB1-192A-F | GCTTACGTTATTGCCACTGGCACCAAC | Construct MoPrb1^D192A H224A S390A^ - mCherry vector |
| MoPRB1-192A-R | GTTGGTGCCAGTGGCAATAACGTAAGC |  |
| MoPRB1-224A-F | GACGGCAACGGTGCCGGTACTCACTGC |  |
| MoPRB1-224A-R | GCAGTGAGTACCGGCACCGTTGCCGTC |  |
| MoPRB1-390A-F | ATCTCTGGCACAGCGATGGCTTCCCCC |  |
| MoPRB1-390A-R | gcccttgctcaccataagcttGGGGGAAGCCATCGCTGTGCCAGAGAT |  |
| MoPRB1-Pro-F | agggaacaaaagctgggtaccCCTATACATGAACCCAGAGC |  |
| MoPRB1-Pro-R | gcccttgctcaccataagcttCACGCGAGCCTCACCGAAA |  |
| MoPEP4-cYFP-F | agggaacaaaagctgggtaccGGAGGCGATAAGGATGATGG | Construct cYFP -MoPep4 vector |
| MoPEP4-cYFP-R | cttgcaggccgggcgaagcttTTTGGCCTTGGCCAGGCCA |  |
| MoPEX14-GFP-F | ACCCAATCTTCAAACTCGAG ATGGCCGACCCTGACAAGAA | Construct MoPex14 -GFP vector |
| MoPEX14-GFP-R | CCCTTGCTCACCATAAGCTTTCGCAACGTGGACCCCTG |  |
|  |  |  |

References

(1) Zheng, W.; Zhou, J.; He, Y.; Xie, Q.; Chen, A.; Zheng, H.; Shi, L.; Zhao, X.; Zhang, C.; Huang, Q.; Fang, K.; Lu, G.; Ebbole, D. J.; Li, G.; Naqvi, N. I.; Wang, Z. Retromer Is Essential for Autophagy-Dependent Plant Infection by the Rice Blast Fungus. *PLoS Genet* **2015**, *11* (12), e1005704.

(2) Liu, X.-H.; Chen, S.-M.; Gao, H.-M.; Ning, G.-A.; Shi, H.-B.; Wang, Y.; Dong, B.; Qi, Y.-Y.; Zhang, D.-M.; Lu, G.-D.; Wang, Z.-H.; Zhou, J.; Lin, F.-C. The small GTPase MoYpt7 is required for membrane fusion in autophagy and pathogenicity of *Magnaporthe oryzae*. *Environ. Microbiol.* **2015**, *17* (11), 4495-4510.
